# The In Vivo Kinetics of RNA Polymerase II Elongation during Co-Transcriptional Splicing

**DOI:** 10.1371/journal.pbio.1000573

**Published:** 2011-01-11

**Authors:** Yehuda Brody, Noa Neufeld, Nicole Bieberstein, Sebastien Z. Causse, Eva-Maria Böhnlein, Karla M. Neugebauer, Xavier Darzacq, Yaron Shav-Tal

**Affiliations:** 1 The Mina & Everard Goodman Faculty of Life Sciences & Institute of Nanotechnology, Bar-Ilan University, Ramat Gan, Israel; 2 Max Planck Institute of Molecular Cell Biology and Genetics, Dresden, Germany; 3 Functional Imaging of Transcription, Ecole Normale Supérieure, Institut de Biologie de l'ENS, IBENS, CNRS, UMR8197, Paris, France; National Cancer Institute, United States of America

## Abstract

RNA processing events that take place on the transcribed pre-mRNA include capping, splicing, editing, 3′ processing, and polyadenylation. Most of these processes occur co-transcriptionally while the RNA polymerase II (Pol II) enzyme is engaged in transcriptional elongation. How Pol II elongation rates are influenced by splicing is not well understood. We generated a family of inducible gene constructs containing increasing numbers of introns and exons, which were stably integrated in human cells to serve as actively transcribing gene loci. By monitoring the association of the transcription and splicing machineries on these genes in vivo, we showed that only U1 snRNP localized to the intronless gene, consistent with a splicing-independent role for U1 snRNP in transcription. In contrast, all snRNPs accumulated on intron-containing genes, and increasing the number of introns increased the amount of spliceosome components recruited. This indicates that nascent RNA can assemble multiple spliceosomes simultaneously. Kinetic measurements of Pol II elongation in vivo, Pol II ChIP, as well as use of Spliceostatin and Meayamycin splicing inhibitors showed that polymerase elongation rates were uncoupled from ongoing splicing. This study shows that transcription elongation kinetics proceed independently of splicing at the model genes studied here. Surprisingly, retention of polyadenylated mRNA was detected at the transcription site after transcription termination. This suggests that the polymerase is released from chromatin prior to the completion of splicing, and the pre-mRNA is post-transcriptionally processed while still tethered to chromatin near the gene end.

## Abstract 2

Kinetic analysis shows that RNA polymerase elongation kinetics are not modulated by co-transcriptional splicing and that post-transcriptional splicing can proceed at the site of transcription without the presence of the polymerase.

## Author Summary

The pre-mRNA emerging from RNA polymerase II during eukaryotic transcription undergoes a series of processing events. These include 5′-capping, intron excision and exon ligation during splicing, 3′-end processing, and polyadenylation. Processing events occur co-transcriptionally, meaning that a variety of enzymes assemble on the pre-mRNA while the polymerase is still engaged in transcription. The concept of co-transcriptional mRNA processing raises questions about the possible coupling between the transcribing polymerase and the processing machineries. Here we examine how the co-transcriptional assembly of the splicing machinery (the spliceosome) might affect the elongation kinetics of the RNA polymerase. Using live-cell microscopy, we followed the kinetics of transcription of genes containing increasing numbers of introns and measured the recruitment of transcription and splicing factors. Surprisingly, a sub-set of splicing factors was recruited to an intronless gene, implying that there is a polymerase-coupled scanning mechanism for intronic sequences. There was no difference in polymerase elongation rates on genes with or without introns, suggesting that the spliceosome does not modulate elongation kinetics. Experiments including inhibition of splicing or transcription, together with stochastic computational simulation, demonstrated that pre-mRNAs can be retained on the gene when polymerase termination precedes completion of splicing. Altogether we show that polymerase elongation kinetics are not affected by splicing events on the emerging pre-mRNA, that increased splicing leads to more splicing factors being recruited to the mRNA, and that post-transcriptional splicing can proceed at the site of transcription in the absence of the polymerase.

## Introduction

The processes of transcription and RNA processing are co-transcriptionally integrated [1]–[7]. A variety of studies have demonstrated that pre-mRNA splicing is coupled to transcription and that intron removal can occur at the site of transcription while the polymerase is still engaged in active transcription. Other mRNA processing events such as 5′ capping and 3′-end formation are also coupled to transcription.

A unique feature of the eukaryotic RNA Pol II is the presence of a long and highly conserved tail at the C-terminus of the large subunit (Rbp1) of the polymerase, termed the CTD (carboxy-terminal repeat domain). It is proposed that the CTD serves as a “jumping board” for protein factors that assemble on the nascent transcript as it emerges from the polymerase [8]–[10]. For instance, certain splicing factors from the SR protein family were found associated with the phosphorylated CTD [11]–[14]. SR proteins are most probably not associated with the transcriptional initiation complex but join in only after the elongation related CTD-phosphorylation has begun. This was demonstrated by showing that RNA Pol II poised at the *Fos* gene promoter is not associated with SR proteins when the gene is in an uninduced state, and only after transcription had begun were SR proteins recruited [15]. Chromatin immunoprecipitation (ChIP) experiments have also demonstrated the association of RNA processing factors with transcribing genes [16]–[19].

Co-transcriptional splicing implies that splicing, at least in part, should occur at the site of transcription. This is supported by kinetic studies showing that splicing is completed within a short ∼5–10 min time-frame from the time of transcription, and these times are not influenced by intron length [20]–[24]. Splicing factors are dynamically recruited to active gene loci [25]–[29]. The kinetics of transcription can influence alternative splice site selection. Polymerase slowdown and pausing affected the pattern of alternative splicing in vivo as seen with a mutant polymerase form that was intrinsically slow-elongating and favored the inclusion of an exon [30]. The current model is that slowing down of the polymerase or polymerase pausing leads to the selection of a weak splice site [1],[31].

In order to examine the opposite relationship, namely whether the kinetics of the elongating RNA Pol II can be influenced by splicing events occurring co-transcriptionally on the associated pre-mRNA, we generated a series of comparable cell lines harboring inducible gene constructs that contain increasing numbers of introns and exons. These genes were stably integrated into a human cell line and formed tandem arrays containing multiple copies of the integrated gene, thereby creating a genomic locus that upon transcriptional activation is easily monitored using live-cell imaging. Such transcription sites have been used previously to analyze the kinetics of recruitment of a variety of nuclear factors to actively transcribing genes (reviewed in [32],[33]). Previous work has demonstrated that the real-time kinetics of RNA Pol II elongation on an active gene can be faster than measured by many biochemical measurements, and is coupled to stochastic pauses of the enzyme [34],[35]. Recent studies have confirmed that Pol II elongation can proceed at rates of 3–4 kb/min [21],[22],[36].

In this study we used the above in vivo approach to examine Pol II elongation rates either on genes that do not contain an intron and therefore are not expected to recruit the spliceosome to the nascent transcript or on genes containing varying numbers of intron/exons and that undergo co-transcriptional splicing. We show that polymerase elongation kinetics are not modulated by splicing events taking place on the emerging pre-mRNA, that increased splicing leads to an increase in the splicing factors recruited to the mRNA, and that post-transcriptional splicing can proceed at the site of transcription without the presence of the polymerase.

## Results

### Generation of Cell Lines with Integrated Intron-Containing Genes

**Figure 1:**
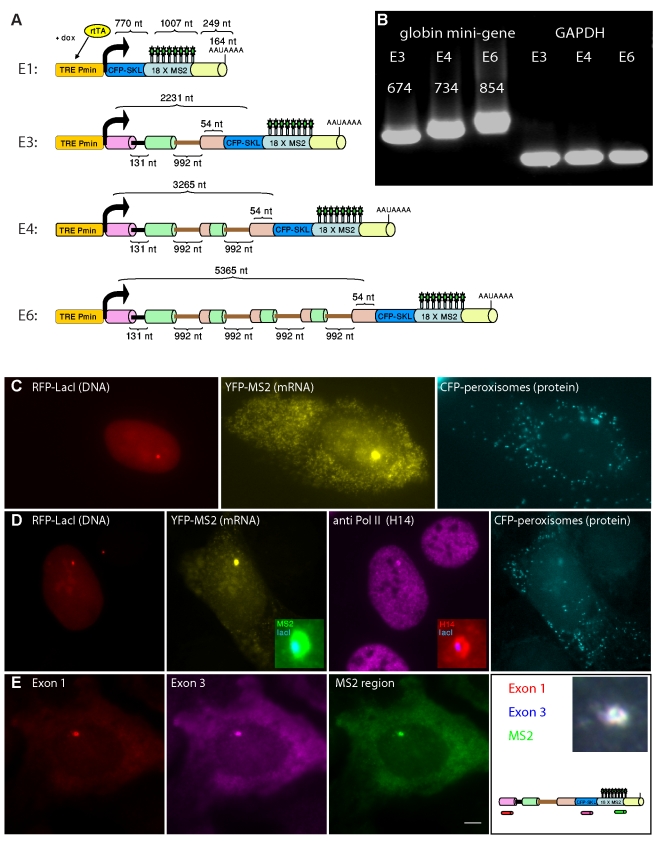
Generating a cell system with comparable genes containing increasing numbers of introns. (A) Gene constructs containing increasing numbers of introns and exons of the human *β-globin* mini-gene were generated and termed E1, E3, E4, and E6. Common to all the genes are: Tet-inducible promoter (orange; Tet responsive element (TRE), minimal promoter (Pmin)) that is induced in the presence of rtTA (yellow) and doxycycline (dox); the genes consist of the *β-globin* intron and exon sequences (exon 1, purple; exon 2, green; exon 3, pink) in frame with a CFP-SKL peroxisomal protein (blue), and a series of 18 MS2 repeats in the 3′UTR (cyan) for the in vivo labeling of the mRNA via binding of YFP-MS2 proteins (green stars). The 3′-end of the gene contains a polyA site. Intron sizes, the total size of the gene, the MS2 repeat region, and the 3′UTR are marked. (B) RT-PCR showing the production of the correctly spliced E3, E4, and E6 mRNAs. GAPDH was used as a control. Sizes(nt) are marked above the bands. (C) E3 cell induced with dox for 12 h (activation times are similar in all experiments unless indicated otherwise) showing the gene locus marked by RFP-LacI (red), the transcribed mRNA at the transcription site and throughout the cell labeled with YFP-MS2 (yellow), and the CFP-SKL protein product in cytoplasmic peroxisomes (cyan). (D) Actively transcribing transcription sites (yellow, YFP-MS2 tagged mRNA) and gene locus (red, RFP-LacI) show the recruitment of endogenous RNA Pol II (purple) by immunofluorescence with the H14 Ab that detects initiating polymerases. Enlargements show the overlay at the transcription site. CFP-peroxisomes are seen in cyan. (E) RNA-FISH showing mRNAs detected throughout an E3 cell with fluorescent probes to the first exon (red), the CFP exon (exon 3, purple), and the MS2 repeats (green). Scheme shows the probe binding sites and the enlargement of the transcription site (bar, 5 µm).

In order to examine the elongation kinetics of RNA Pol II during splicing, we generated the following gene constructs. The first construct consisted of the human *β-globin* mini-gene with three exons and two introns and was therefore designated E3 (Figure 1A) [37],[38]. Exon 3 was truncated and fused in-frame to a cyan fluorescent protein (CFP) coding region containing the peroxisomal targeting tripeptide Ser-Lys-Leu (SKL) in its C-terminus [39], thereby generating cyan fluorescing peroxisomes throughout the cytoplasm, indicative of productive gene expression. Immediately downstream were a series of 18 MS2 sequence-repeats. When transcribed, these repeats form 18 stem-loops in the mRNA and provide high-affinity binding sites for the MS2 coat protein [40],[41]. The detection of these mRNAs in vivo is accomplished by the binding of dimers of the YFP-MS2 coat-protein to each stem-loop, thereby forming fluorescently labeled mRNAs [42]. This occurs simultaneously during active transcription [34],[41].

*β-globin* was chosen as a model gene since it has been extensively used for in vitro splicing assays [43]. In this manner we could use a system that is close to the endogenous state of a gene. The first intron of *β-globin* is short (131 nt), while most typical genes have a long first intron [44]. The length of the second *β-globin* intron is 992 nt, which is close to the median size of internal introns (∼1,400 nt) [44]. Thereby, we could use repeats of the second intron to generate additional genes containing increasing numbers of introns.

In order to compare between genes, we generated additional gene constructs (Figure 1A): (a) E1 – an intronless gene in which exons 1, 2 and their introns were deleted, thereby leaving part of exon 3 + CFP-SKL to serve as a single exon; and (b) E4, in which intron 2, flanked by the splice sites and part of exons 2 and 3, was duplicated, thereby forming a gene construct with 4 exons and 3 introns. The E6 construct (containing another two repeats of intron 2) will be described later on. All the constructs were under the inducible control of the Tet-On system. In the presence of the rtTA (Tet-On) transactivator and the addition of doxycycline (dox) to the medium, transcription was induced.

Each of the constructs was stably integrated into human U2OS Tet-On stable cells. The different sized gene constructs produced the correctly spliced mRNAs as seen by RT-PCR on mRNA extracted from the three cell lines following transcriptional activation by dox: E3  =  2,279 nt (unspliced 3,402 nt), E4  =  2,321 nt (unspliced 4,436 nt) (Figure 1B). The E1 mRNA (1,941 nt) did not undergo splicing. The genes were co-integrated with a plasmid containing 256 lac operator repeats (*lacO*) in order to enable the detection of the genomic locus of integration [29],[45]. Using a fusion protein of the lac repressor protein (LacI), e.g. RFP-LacI, the genomic locus was tagged, allowing the detection of the integration locus independent of the transcriptional status of the gene. Selection of positive stable clones was performed after induction with dox and identification of cytoplasmic CFP-peroxisomes in all cell clones (Figures 1C and S1), altogether indicating accurate transcription, splicing, export, and translation. Co-integration of the 256 *lacO* repeats was verified by the expression of RFP-lacI and the detection of single genomic integration sites (Figure 1C). YFP-MS2-labeled mRNAs were detected at the transcription site, as well as in the nucleoplasm and the cytoplasm as expected (Figure 1C). Several stable clones were kept for each gene, and we proceeded to work with three cell lines designated E1, E3, and E4, in accordance with the integrated gene.

Stable transfection results in the integration of multiple copies of the gene construct at one genomic locus that is termed a “tandem gene array,” which is useful for live-cell imaging experiments of active transcription sites [32]. We confirmed that RNA Pol II in both active phosphorylated forms was recruited to the transcription sites using the H14 (CTD phosphorylated on serine 5), H5 (CTD phosphorylated on serine 2), and 8WG16 antibodies (specific to the CTD repeats) (Figures 1D and S2A,B). RNA FISH with probes to different regions in the pre-mRNA and mRNA were used to detect the distribution of the mRNAs in the cells following dox induction. Probes to exon 1, the CFP coding region (exon 3), or the MS2 region (3′UTR) of the E3 gene, detected the active transcription sites as well as cytoplasmic mRNAs in induced cells, and some nuclear signal, suggesting robust nucleo-cytoplasmic transport of the mRNAs to the cytoplasm (Figure 1E). No RNA FISH signal was seen in un-induced cells. Video S1 shows the activation of transcription, nucleo-cytoplasmatic transport of the tagged mRNA, and the appearance of the CFP peroxisomes in the cytoplasm. Altogether, we generated a series of cell lines in which the kinetics of transcription could be monitored on integrated genes in real-time.

### Co-Transcriptional Splicing Occurs at the Site of Transcription

**Figure 2:**
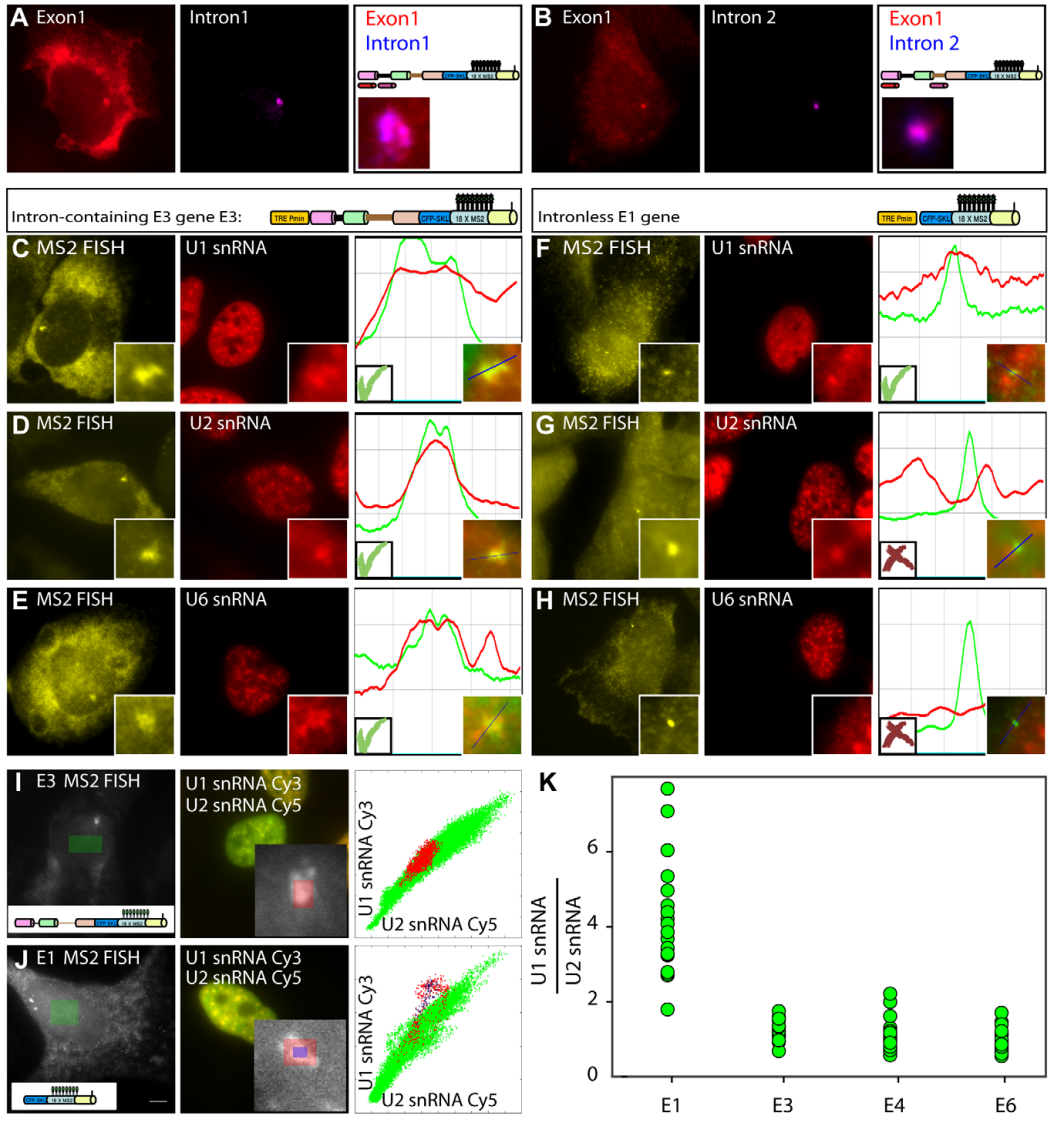
Co-transcriptional splicing occurs at the site of transcription. (A) A probe to the first intron or (B) the second intron (purple) was detected in an E3 cell only at the transcription site while the mature mRNA was detected throughout the cell (probe to exon 1, red). Schemes on the right show the probe binding regions and an enlargement of the colocalized signals at the transcription site. (C–H) The recruitment of the core pre-mRNA splicing machinery to active transcription sites was examined in E3 (left column) and E1 (right column) cells. RNA-FISH with an MS2 probe (yellow) shows the active transcription sites together with snRNA probes in red: (C and F) U1 snRNA, (D and G) U2 snRNA, and (E and H) U6 snRNA. Enlargements show the signals at the transcription sites. Plots on the right depict the degree of colocalization of the signals at the transcription site across the blue line. (I) RNA-FISH signal of U1 and U2 snRNAs (middle) at the active transcription site (seen with the MS2 probe, left) of an E3 cell and (J) an E1 cell were measured. The green box shows a random nucleoplasmic area, and the red and blue boxes show the area of the transcription site. The plot shows the correlation of the U1 and U2 snRNA signals at the transcription site (red and blue dots) in comparison to the nucleoplasm (green dots) (bar, 5 µm). (K) The ratio between U1 and U2 snRNA signals measured by RNA-FISH, at the transcription sites of all genes. The data in (I), (J), and (K) show a constant correlation in the intron-containing genes, and no correlation in the intronless E1 gene, meaning that U1 and not U2 is recruited even to an intronless gene.

Probes to the first or second intron regions displayed signal only at the sites of transcription without additional signal in the nucleoplasm or cytoplasm (Figure 2A,B), showing that the splicing of introns occurred at the site of transcription. In order for co-transcriptional splicing to occur, splicing factors must be recruited to the pre-mRNA either by diffusing from the nucleoplasm or possibly via the CTD of RNA Pol II. A previous proteomic study immunopurified more than 100 proteins associated with RNA Pol II in a non-transcribing extract [46], among them U1 snRNP components and factors of the SR family. In order to assay the recruitment of splicing factors in vivo we examined whether different factors were enriched on the active E3 and E4 gene arrays, using immunofluorescence to endogenous proteins or expression of fluorescently-tagged versions of these proteins. snRNPs, splicing factors, and other RNA processing factors were detected (Figures S2C–J and S3). These data are summarized in Table 1. In addition, using RNA FISH probes specific to the different snRNAs we could detect U1, U2, U4, U5, and U6 snRNAs at the site (Figures 2C–E, S2D–E, and Table 1). Not all molecules expected to be present at the transcription sites were detected, e.g. PSF, p54^nrb^, and 7SK-RNA (Figure S3B,C, and F). We conclude that co-transcriptional splicing is occurring on the pre-mRNAs associated with the active transcription sites, and that the mRNA processing factors are recruited either via association with the transcribing polymerase or directly with the nascent mRNAs.

**Table 1:** Examination of the proteins and RNAs that are recruited to intron-containing and intronless genes.

Recruited to Intron-Containing Transcription Sites (E3/E4/E6)	Not Recruited to Intron-Containing Transcription Sites (E3/E4/E6)	Recruited to an Intronless Gene (E1)	Not Recruited to an Intronless Gene (E1)
Active polymerase II (H14)	PSF	Active polymerase II (H14)	U2 snRNA
Active polymerase II (H5)	p54^nrb^	Active polymerase II (H5)	U4 snRNA
Polymerase II CTD repeats (8WG16)	7SK RNA	Polymerase II CTD repeats (8WG16)	U5 snRNA
2,2,7-trimethylguanosine (m_3_G) (anti-TMG)		2,2,7-trimethylguanosine (m_3_G) (anti-TMG)	U6 snRNA
U1 snRNA		U1 snRNA	U2AF65
U2 snRNA		U1A	
U4 snRNA			
U5 snRNA			
U6 snRNA			
U1A			
U2AF65			
SF2/ASF			
SC35			
9G8			
TLS			
PTB			
Cyclin-T1			

### The U1 snRNP Is Recruited to an Intronless Gene

We then tested for the recruitment of the spliceosomal U snRNPs to the intronless E1 gene. Using an antibody that recognizes the 2,2,7-trimethylguanosine (m_3_G) cap of snRNA (anti-TMG) we could detect TMG staining on the transcription site of E1 (Figure S4A). RNA FISH showed that U1 snRNA was present on the site, as well as the U1A protein (Figures 2F and S4B). However, all other snRNAs or U2AF65 were not found on the E1 sites (Figure 2G–H, S4C–E and Table 1).

The recruitment of U1 snRNA to the E1 and E3 genes was measured and compared by correlating the intensity of the U1 and U2 signals on the transcription site to the same two signals within the nucleoplasm. In the nucleoplasm we expected to find co-localization of U1 and U2 snRNA signals, since spliced genes are predominant. In the case of the E3 and E4 genes, the U1/U2 correlation at the transcription site was the same as in the nucleoplasm (Figure 2I). However, in the case of the E1 gene a different correlation was observed between U1 and U2 at the transcription site compared to the U1/U2 nucleoplasmic ratio (Figure 2J). We then quantified this recruitment. The measured ratio of U1 and U2 snRNA signals on the different genes demonstrated a constant ratio between U1 and U2 on the intron-containing genes (E3, E4, and E6), while on the E1 gene the presence of U1 snRNA was dominant and U2 was not present (Figure 2K). Interestingly in a recent study, the association of U1 components on a gene construct in which splicing did not take place due to mutations in the 5′- and 3′-splice sites was demonstrated [47]. We therefore conclude that U1 snRNPs are associated with the transcriptional machinery on all transcribing genes, no matter whether the pre-mRNA contains introns or not, probably through interactions with the CTD of the active polymerase [46],[47].

### The Effect of Co-Transcriptional Splicing on Pol II Elongation Kinetics

**Figure 3:**
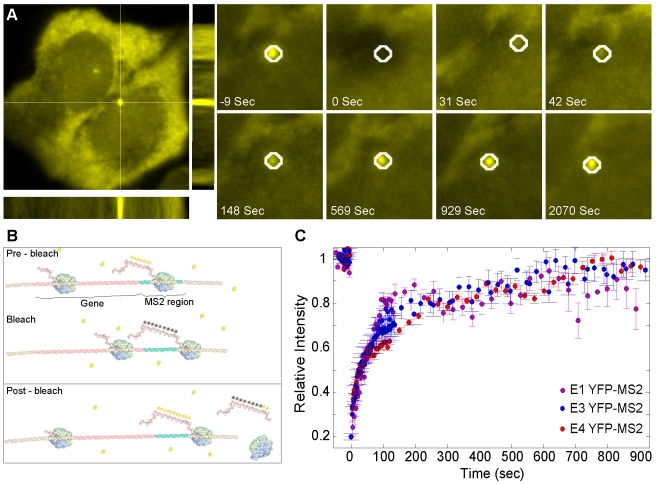
Comparison of transcriptional kinetics on active E1, E3, and E4 transcription sites. (A) An active transcription site (YFP-MS2, bottom cell) was photobleached and the fluorescence recovery was tracked over time (frames on right). These experiments were performed on a 3D FRAP system (XZ and YZ representations are seen under and to the right of the original image, respectively). (B) Scheme of the elongation assay showing bleaching of the YFP-MS2 proteins (yellow) bound to the mRNA and the recovery of fluorescence as new polymerases elongate through the MS2 region. (C) Recovery curves of the YFP-MS2 FRAP measurements performed on E1, E3, and E4 transcription sites. Average of *n*>10 experiments for each cell type with SDE.

After showing that pre-mRNA splicing is taking place at the site of transcription in the E3 and E4 genes, we examined whether polymerase elongation kinetics differed between the intronless and intron-containing genes. We used the previously described in vivo elongation assay [34],[35],[41] to measure the kinetics of RNA Pol II on the three genes. In this assay, active transcription sites are detected with the YFP-MS2 coat protein. This fluorescent signal at the transcription site consists of the steady state kinetics of mRNA synthesis as well as mRNA release from the site. Using a fluorescence recovery after photobleaching (FRAP) approach, the YFP-MS2 signal at the transcription site is photobleached and the recovery of the YFP-MS2 signal is monitored over time. Fluorescence recovery signifies the generation of new MS2 stem-loops in the nascent transcripts and their binding by fluorescent YFP-MS2 molecules entering from the surrounding nucleoplasm (Figure 3A and 3B).

This same approach was implemented on the E1, E3, and E4 genes. The cell lines were co-transfected with RFP-LacI and YFP-MS2, and transcription was induced by dox for at least 6 h until transcription reached steady state. Active transcription sites at steady state did not show any significant difference in YFP-MS2 intensity for at least 20 min. Using a 3D-FRAP microscope system we could photobleach the transcription site (YFP-MS2 signal) and continue to detect the gene locus using the RFP-LacI DNA label. The advantage of using the 3D-FRAP microscope is that rapid 3 dimensional (3D)-imaging over time (4D) could be performed. Thereby, the signal of the whole nuclear volume could be collected with no effect on the measurements, and motion of the transcription site was easily tracked (Figure 3A and Video S2). This is in comparison to most confocal microscopes that collect the information only in 2 dimensions, and therefore measurements would be sensitive to any DNA motion in the *z*-axis.

Transcription sites were followed for extended periods (up to 30 min). The YFP-MS2 signal intensity on the transcription sites was quantified using a tracking algorithm. We measured recovery kinetics on the transcription sites of all the three gene-types in order to examine whether different elongation kinetics could be identified. We compared a polymerase passing through the MS2 region of an intronless E1 gene to a polymerase moving through the MS2 region of the intron-containing E3/E4 genes that undergo co-transcriptional splicing (after the exons and introns are transcribed). The FRAP recovery curves measured on the E1, E3, and E4 genes all showed similar recovery kinetics (Figure 3C), indicating that the polymerase transcribing the MS2 region was not affected by the presence of active splicing from the upstream sequences. We conclude that upstream splicing events do not necessarily affect the elongation kinetics on our model genes. However, it is possible that other differences such as in intron sequence or intron size (e.g. the *β-globin* first intron is shorter than average) might cause some effect on elongation. These experiments were performed on several microscope stations to verify reproducibility (Figure S5). Altogether, these measurements indicate that polymerase transcription rates are not modulated by the co-transcriptionally assembled spliceosomal machinery.

### Increasing Intron Numbers Leads to an Increase in Spliceosome Component Recruitment

We examined a similar gene construct in which additional introns and exons were very closely spaced. Thereby, we expected transcription to end before the completion of splicing of all introns, which would allow the further examination of elongation kinetics. The E6 gene was generated using the E4 construct and consisted of 6 exons and 5 introns by using duplications of intron 2 (Figure 1A). In this manner, we generated a gene with 6 exons (close to the 8 exon median size of genes [44]). The E6 mRNA (2,437 nt) was correctly spliced (unspliced 6,536 nt, Figure 1B), the CFP-SKL peroxisomal protein was expressed normally (Figure S1 and Video S1), and similar recruitment of splicing factors was observed (unpublished data).

**Figure 4:**
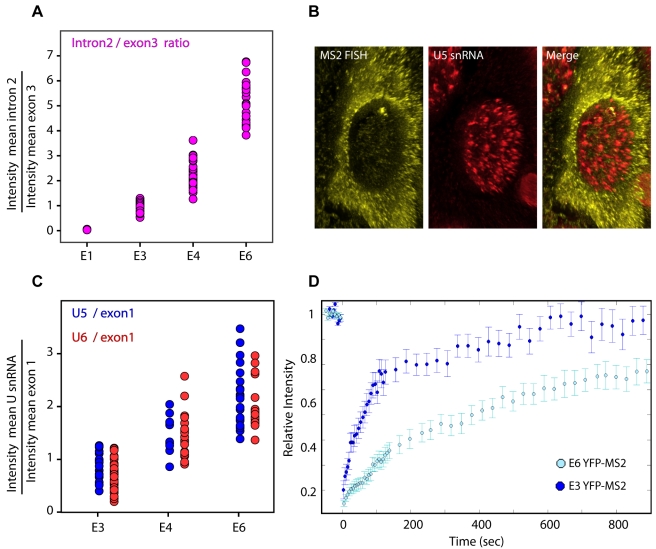
Increased spliceosome recruitment and change in transcriptional kinetics in relation to increasing intron numbers. (A) Quantification of the RNA-FISH signals showing the ratio of the second intron to the last exon (CFP region) on the different transcription sites (all normalized to the E3 ratio). (B) A 3D representation of the deconvolved images of an RNA-FISH experiment in which MS2 mRNA (in yellow) and U5 snRNA (in red) were detected. The signals at the transcription site were measured for both channels. The elongated form of the signal is due to the 3D projection of the images. (C) Quantification of the RNA-FISH signal showing the ratio between transcription site associated U5 (blue dots) or U6 (red dots) snRNAs in comparison to exon 1 on the E3, E4, and E6 genes. Each dot represents a transcription site ratio. (D) FRAP recovery curves of YFP-MS2 on E3 versus E6 genes with standard error bars (*n*>10).

First, we verified that there was an increase in accumulation of introns at the transcription site of the E6 gene compared to the other genes, as expected. 3D volumes of RNA-FISH labeled cells were collected, deconvolved, and analyzed. We calculated the fluorescence ratio of intron 2 in comparison to the last exon (CFP exon), in all the cell lines. Higher levels of introns generated were detected on the E6 gene (Figure 4A) compared to the other genes. As expected, there was a gradual accumulation of intron 2 at the active transcription sites in dependence to the number of introns in each gene.

We next asked whether there would be increased accumulation of the splicing machinery on genes containing more introns. We therefore quantified the relative amounts of the splicing machinery (U snRNAs) that accumulated on each transcript using 3D volumes of RNA-FISH labeled cells (Figure 4B and 4C). Quantification of the fluorescence ratio of U5 or U6 snRNAs in comparison to the first exon present in each nascent transcript demonstrated a higher accumulation of snRNAs that was dependent on the number of introns in the gene. This indicated that the addition of exons resulted in an increase in the recruitment of the splicing machinery per transcript. These data also imply that the elongation machinery does not wait for each splicing event to complete before moving forward on the gene, but rather continues to elongate while more spliceosomal units accumulate on the transcript.

### The E6 Gene Exhibits Slower Recovery Kinetics

Next, polymerase kinetics on the E6 gene were measured. Interestingly YFP-MS2 FRAP curves showed significantly slower recovery kinetics compared to the previous measurements on the E1, E3, and E4 genes (Figure 4D). In order to verify that this was not a clone-related difference, and that the measurements were not affected by the number of gene units in the tandem array or from the DNA integration site, we compared two different E6 clones, E6-22 (40 gene copies) to clone E6-3 (9 gene copies). The copy number of gene integration in each cell line was quantified by real-time PCR on genomic DNA. We found that both E6 clones recovered with identical and relatively slower kinetics (Figure S6A and S6B).

To identify the cause for the kinetic difference between the genes, we analyzed the measured FRAP recovery curves. Two previous studies have used this MS2 FRAP approach to measure Pol II transcription rates on specific genes [34],[41]. The assay measures the recovery of fluorescence as the polymerase transcribes the MS2 region right after the exons and introns are transcribed (Figure 3B). The recovery signal shows bi-phasic kinetics (Figure S7A), which best fit to two exponentials. This implies that the measurements are detecting two processes that are occurring in parallel, which require the interpretation of the significance of each phase in the curves. The abovementioned studies have used extensive modeling to study such FRAP curves and demonstrated that the fast phase of fluorescence recovery is generated by the actively elongating polymerases. The slow phase of recovery was explained as either a population of polymerases in a paused state along the gene [34] or a termination delay [41]. The slow kinetics on the E6 gene could therefore be caused either by polymerase slowdown or by retention of the mRNA after termination. This was further tested.

**Figure 5:**
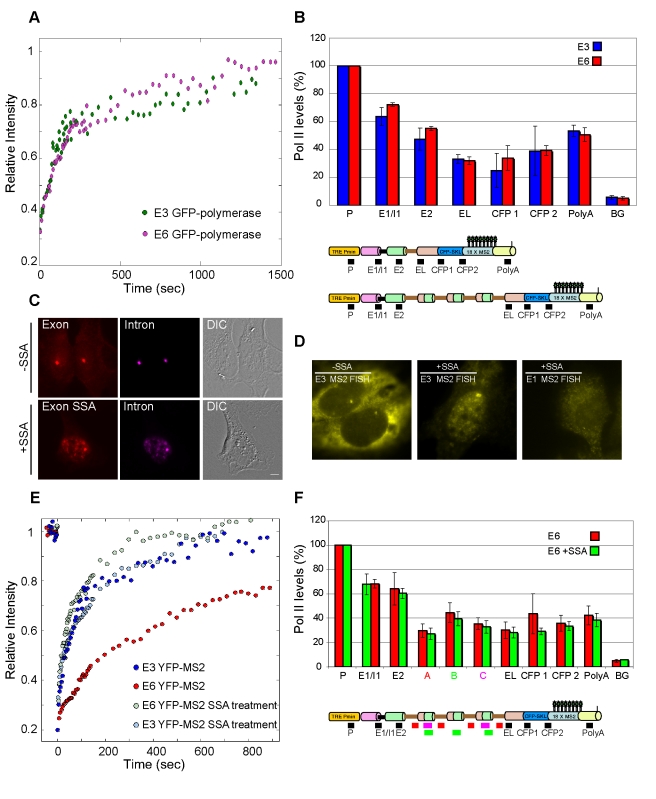
Dynamics of E6 mRNA are affected by active splicing and not by polymerase kinetics. (A) FRAP recovery curves of transfected GFP-Pol II recruited to the E3 and E6 genes. (B) ChIP analysis of Pol II distribution along the E3 (blue bars) and E6 (red bars) genes. Black boxes under the schemes indicate primer positions. The data were normalized to the promoter by setting the promoter value at 100%. A *t*-test for each primer set showed no significant differences (*p*>0.05). (C) Inhibition of splicing by Spliceostatin (SSA) for 9 h showed that unspliced pre-mRNA (intron probe, magenta) was distributed throughout the cell and in speckles (RNA-FISH on E6 cells) and was retained in the nucleus (bottom), whereas in untreated cells pre-mRNA was detected only at the site of transcription (intron probe, magenta; exon probe, red) (bar, 5 µm). (D) SSA treatment (6 h) caused the nuclear retention of intron-containing E3 mRNA (middle) versus the regular nucleo-cytoplasmatic dispersal of E1 intronless mRNA (right). Left, untreated E3 cell. RNA-FISH was performed to the MS2 region of the mRNAs (yellow). (E) FRAP recovery curves of YFP-MS2 at the transcription sites of untreated and SSA-treated E3 and E6 cells. (F) ChIP analysis of Pol II distribution along the E6 gene in untreated (red bars) and SSA-treated (green bars) cells. Boxes under the schemes indicate primer positions. The data were normalized to the promoter by setting the promoter value at 100%. A *t*-test for each primer set showed no significant differences (*p*>0.05).

We first examined whether the kinetic effect was due to a change in polymerase activity. FRAP of the active polymerase transcribing on the E3 and E6 genes was analyzed using a transfected GFP-Pol II that was recruited to the active genes. Similar FRAP recovery kinetics were measured on both genes (Figure 5A). This measurement differs from the above measurement of YFP-MS2 FRAP recovery. While the latter measures elongation through the MS2 region only, Pol II FRAP encompasses the whole polymerase transcriptional process on the gene, ranging from initiation through elongation and termination [34]. The Pol II FRAP measurements (Figure 5A) indicated that the polymerases on E3 and E6 genes were functioning with similar kinetics with regard to the general transcriptional flow. However, since only the very slow recovery phase is attributed to the elongating phase of the polymerase (consists of only <30% of the signal) [34], it is difficult to resolve between the polymerase kinetics on E3 and E6. Therefore, we obtained chromatin immunoprecipitation (ChIP) information to compare the distribution of Pol II along the genes, which might detect regions of paused polymerases. Using pull-down of the polymerase with an anti-Pol II antibody followed by real-time PCR on 7 different gene regions, we corroborated the polymerase FRAP findings and demonstrated that there was no statistical differences in the density of Pol II along the two genes (Figure 5B). Additionally, from these data we can learn that the highest Pol II density is found at the promoter and the polyA site, which is in agreement with other studies [16]. Altogether, these results showed that the detected kinetic change in E6 was not directly connected to the polymerase.

### Splicing Inhibition Relieves the Slow Kinetics on the E6 Gene

To examine whether the E6 kinetic delay was due to ongoing splicing, we used two splicing inhibitors, Spliceostatin A (SSA) [48] and Meayamycin [49]. SSA and Meayamycin are inhibitors of pre-mRNA splicing via SF3b binding, which binds to the intron branch-point and acts as a subcomplex in the U2 snRNP. SSA treatment caused the accumulation and retention of unspliced pre-mRNA in the nucleoplasm, which continued to accumulate in nuclear speckles, as detected by RNA-FISH with an intron probe (Figure 5C), as previously described [48]. We now show this same effect also for Meayamycin (Figure S8A). Interestingly, the dispersal of the intronless E1 mRNA was not affected, and it did not accumulate in speckles (Figure 5D). Then we measured YFP-MS2 recoveries by FRAP on the E6 gene in untreated cells as described above and incubated the same transcribing cells with either SSA or with Meayamycin. Under both treatments the slow kinetics of E6 were transformed to the regular kinetics of E3/E4/E1 genes (Figure 5E and S8B), whereas splicing inhibition did not cause any effect on the kinetics of the E3 gene (Figure 5E). On the other hand, FRAP of GFP-Pol II in the presence of SSA on the E6 gene (Figure S8C), and ChIP experiments in the presence of SSA (Figure 5F) did not show any change in kinetics. These results demonstrate that splicing inhibition did not modify Pol II distribution or kinetics along the genes. This emphasized that the changes in kinetics between E6 and E3 genes were due to active splicing events and were not connected to the elongation activity of the polymerase.

### RNA Splicing Can Lead to Retention of the Pre-mRNA on the Transcription Site

**Figure 6:**
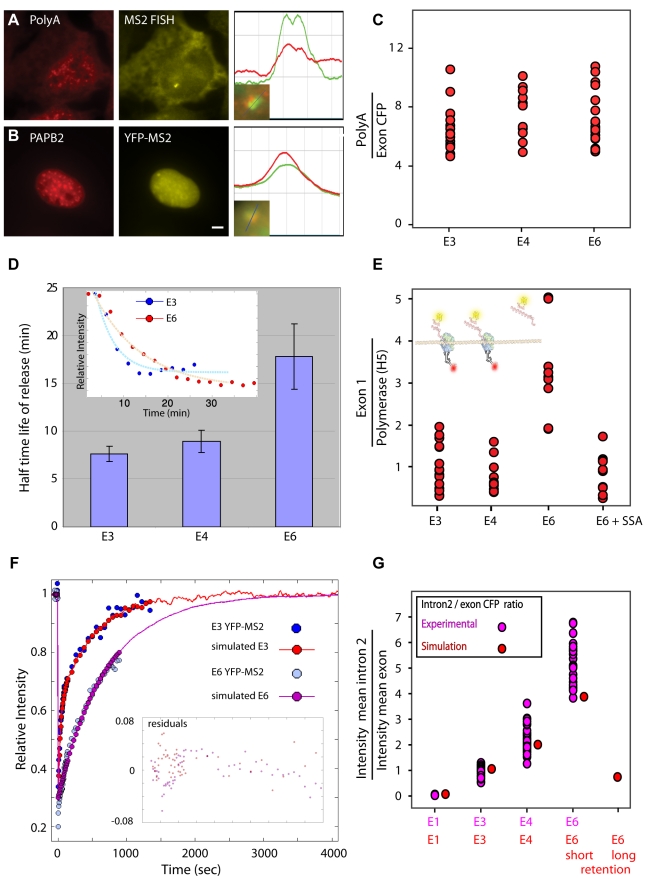
RNA splicing leads to retention of the pre-mRNA on the transcription site. (A) RNA FISH in an E3 cell with a polyT probe (red) and an MS2 probe (yellow) showing that polyadenylated mRNAs were formed and were present on the transcription sites. (B) Recruitment of GFP-PABP2 (pseudocolored red) to the transcription site labeled with YFP-MS2 (yellow). Plots on the right in (A) and (B) depict the degree of colocalization of the signals at the transcription site across the line (bar, 5 µm). (C) The ratio of the RNA-FISH signal of polyA to the last exon on the E3, E4, and E6 active transcription sites, showing no aberrant accumulation of transcripts without polyA signal. (D) Half-time of release of mRNA from transcription sites following actinomycin D treatment (time measured from ActD addition) in E3 (*n*  =  7) and E4 (*n*  =  7) cells compared to E6 cells (*n*  =  8). Inset shows two sample time-series of transcription site inactivation and the exponential fit from which half-times of clearance were calculated (blue, E3; red, E6). (E) The ratio between the polymerase signal and the first exon on the E3, E4, E6, and SSA-treated E6 genes. Each red dot represents the ratio of intensities measured on a single transcription site. The scheme at the top shows the polymerase antibody (red) and the mRNA (FISH exon 1, yellow). (F) YFP-MS2 FRAP data and the fitted simulation of the E3 and E6 genes. The calculated residuals of the fit are presented in the inset. (G) RNA-FISH ratio of intron 2 to the last exon at the transcription site in E1, E3, E4, and E6 cells. The experimental data points (pink, from Figure 4A) were compared to the simulation output (red) of the same experiment. Simulations were performed with either short (50 sec, E3 kinetics; E1, E3, E4, E6) or long (10 min, E6 kinetics; E6 rightmost point) transcript retention times.

The above experiments suggested that the slow recovery FRAP curves of the YFP-MS2 experiments were due to a delay of the nascent transcripts on the E6 sites caused by splicing but without connection to the polymerase itself. First, we verified that the full transcripts were 3′-end processed and were present at the site of transcription of all the genes prior to release. This was shown by the recruitment of GFP-PABP2 to the active transcription sites and by the presence of polyA tails as seen by RNA-FISH (Figure 6A and 6B). RNA-FISH measurements of the ratio of polyA signal to the last exon (CFP) on the sites showed that a constant ratio was retained on all the transcription sites (Figure 6C), meaning that there was no accumulation of mRNAs lacking polyA on the different genes and no change in polyadenylation levels. This suggested that there was an accumulation of transcripts that were not attached to Pol II, at the transcription sites.

To characterize the delay time of the mRNAs on the E6 transcription sites, we next examined the kinetics of mRNA release from the gene. Actinomycin D (ActD) was added to actively transcribing cells containing YFP-MS2 tagged transcription sites. The “shut-down” of the transcription sites was monitored over time under the microscope. Indeed, slower clearing of the mRNAs from the gene was observed in E6 cells (t_1/2_  =  17 min) compared to E3 cells (t_1/2_  =  7 min) and E4 (t_1/2_  =  8.9 min) (Figure 6D, Video S3). This experiment correlates with the YFP-MS2 FRAP measurements (Figure 4D) showing slower mRNA kinetics on the E6 gene.

Finally, to demonstrate that only the mRNAs disconnected from the polymerase were stalled on the E6 gene, we quantified the intensity ratio of the polymerase signal and the mRNA signal on the transcription sites using anti-Pol II immunofluorescence and RNA-FISH to the first exon. If indeed each mRNA is attached to one polymerase, we would expect a constant ratio for all cell lines. However, we found that the proportion of mRNA to Pol II on E6 transcription sites was three times higher than on E3 and E4 sites (Figure 6E). When SSA was added and splicing was inhibited, the mRNA/Pol ratio on the E6 sites was reduced to the levels of E3 and E4 sites, meaning that under conditions of splicing inhibition, the unspliced E6 pre-mRNAs were being released without stalling similarly to the E3 and E4 mRNAs. These experiments implied that in a situation like the E6 gene which contains closely spaced introns, and in which the polymerase has finished transcribing yet the pre-mRNA is not fully spliced, the mRNA is retained at the transcription site until the completion of splicing.

To better understand the given observed results we simulated the transcriptional process using a stochastic Monte-Carlo simulation (Figure S9A and Video S4). The simulation mimicked the transcriptional process based on the transcriptional models described in the previous studies [34],[41]. Kinetic parameters were obtained by fitting the simulations to the experimental FRAP curves of E3 (Figures 6F). See Material and Methods for explanation of parameter scanning. The parameters that gave the best fit were: an elongation speed of 3.6 kb/min, agreeing with previous measurements [21],[22],[36]; ∼42% of the polymerases had entered a paused state for an average time of 6 min [34]; and an average retention time (after end of elongation) of 50 s before release from the transcription site (Figures S9 and S10) [41]. We found that the number of genes in the array and the initiation rate had no effect on the measured kinetic results, due to the normalization of the data to the steady-state pre-bleach signal. This observation was confirmed experimentally by measuring FRAP experiments with low amounts of DRB to abrogate elongation. Indeed, no kinetic effect on E3 or E6 YFP-MS2 FRAP recoveries was observed (Figure S11), while the addition of SSA even in the presence of DRB transformed E6 kinetics to E3 kinetics, signifying the effect of splicing on the retention of mRNA at the site of transcription.

We then searched for a reliable fit to the E6 data by keeping the kinetic parameters of E3 and changing only the time-scale of transcript release. We found that the average time for a transcript to remain at the E6 site of transcription was on average ∼10 min (Figure 6F). This is in agreement with the actinomycin D experiment that showed a 10 min difference in transcript clearance times between E3 and E6 (Figure 6D). We also used the simulations to calculate the ratio of polymerases to nascent transcripts on the active genes, and compared to the experimental data. The simulation showed a 3-fold increase in this ratio on the E6 gene, similar to the result obtained with quantitative FISH (Figure 6E).

We then used this simulation to examine the intron/exon ratio on the different genes. In this experiment (Figure 4A) we found a gradual increase in intron signal at the transcription sites that correlated to the number of introns in each of the genes. The simulation output was compared to these RNA-FISH measurements. The simulation provided a ratio between the number of polymerases/transcripts present on the duplicated intron 2 region and the number of transcripts that had already passed through the CFP region. Simulations were run for the different genes with the abovementioned kinetic parameters assigned to E3 (short retention time). Since this experiment follows the polymerase moving along the genes ( = elongation), we expected an increase in the intron/exon ratio from E1 to E6 genes. Indeed, the simulation showed the same increment in intron/transcript ratio as seen in the experimental results (compared in Figure 6G). However, when the calculated long retention kinetics at the end point of the gene was included in the simulation of E6 kinetics, we obtained an output that showed a low intron/exon ratio, which did not agree with the experimental data (Figure 6G). Therefore, the continuous flow of intron generation observed with the simulation, in contrast with the slow kinetics of the transcript, imply that the release time of the transcript from the gene site depends on splicing and not on the polymerase kinetics.

## Discussion

Gene expression profiles are influenced by the presence or lack of an intron. It has been known for many years that the addition of an intron into the cDNA of a gene sequence enhances its expression [50]. Indeed, the act of intron removal can have effects on the initiation of transcription, mRNA polyadenylation, mRNA export, and the cytoplasmic fate of the transcript such as translation, mRNA localization, and mRNA turnover [51]. For instance, several studies have demonstrated that the presence of an intron in the pre-mRNA and subsequent splicing can elevate the rates of mRNA export and thereby the accumulation of message in the cytoplasm [52]–[54]. Hence, the presence of an intron exerts an overall positive influence on gene expression.

How does intron removal affect the levels of transcription and mRNA production? Some level of regulation can be found on the DNA itself, for instance by intronic sequences that modulate nucleosome assembly in the vicinity of the promoter [55]. Splicing signals in the 5′ promoter-proximal intron can stimulate polymerase II initiation, processivity, and recruitment of transcription factors [56]–[59], and complexes containing transcription and splicing factors have been identified [60]. Nonetheless, the increase of Pol II activity on intron-containing genes versus intronless genes was modest [56]. Recently, the depletion of the SR protein SC35 in cells was shown to attenuate the elongation of Pol II within the gene sequence [61].

In this study we wished to examine the effects of splicing on Pol II elongation in vivo, independent of promoter influences and transcriptional initiation events. Therefore, all the gene constructs were controlled by the same inducible promoter, and elongation kinetics were examined within the gene as far as possible from the initiation site. Elongation rates were measured immediately downstream of the region transcribing the exon-intron-exon elements, or in other words we could monitor the polymerase speed as it moved along the DNA after the splicing codes in the pre-mRNA had already been synthesized. This meant that polymerase movement towards the 3′-end of the gene was occurring simultaneously with splicing events accompanying the newly synthesized pre-mRNA. We measured intron accumulation on the active transcription sites that was dependent on intron numbers.

The presence of various splicing machinery factors at the transcription sites was detected only when the gene loci were actively transcribing. Furthermore, RNA-FISH to intronic and exonic sequences demonstrated the presence of the introns only at the sites of transcription and not in the nucleoplasm, signifying that this is the location of the removal of these introns. However, when splicing was inhibited with Spliceostatin or Meayamycin, intron-containing pre-mRNA could be detected also throughout the nucleus, in particular within nuclear speckles, as previously described for SSA [48]. The intronless E1 mRNA was not affected by these treatments. Splicing factors associated with the actively transcribing genes belonged to the U snRNP family, including snRNAs (U1, U2, U4, U5, U6) and protein components (U1A, U1-70K, U2AF65). Also SR proteins such as SC35, SF2/ASF, and 9G8 were recruited to the active transcription sites upon activation. Other factors detected at the sites were the transcription/splicing-related protein TLS/FUS, splicing regulator PTB, the elongation regulator cyclin T1, and polyA-binding protein 2 (PABP2). However, factors like PSF and p54^nrb^ [62] previously demonstrated to be associated with the Pol II CTD [63] were not detected at the transcribing genes, nor were they pulled down with a purified polymerase [46]. Distinguishing between the two approaches might hint to transcription factor dynamics. While purified protein complexes can give an idea of the “presence” of a factor in a transcription complex, the imaging assays can indicate the longevity of “residence time” at the active gene. The fact that these factors were not identified accumulating on the transcribing gene arrays using antibodies to the endogenous proteins could indicate short-lived or low-affinity interactions with other factors that do associate with the actively transcribing gene.

In this study we examined spliceosomal recruitment to comparable intron-containing genes as well as an intronless gene. As anticipated, spliceosomal factors were not found on the active transcription site of an intronless gene, except for components of the U1 snRNP. These data are in agreement with a recent report that quantified the recruitment of splicing factors to an intron-containing gene in which the splice sites were mutated and therefore did not undergo splicing [64]. In that study the recruitment of U1 snRNA and U1-70K were detected on the gene. In our analysis the intronless E1 gene does not contain any splice sites and therefore strengthens the notion that U1 snRNP is associated with the CTD of Pol II by default and presumably accompanies the polymerase during transcription in order to scan for the presence of a 5′ splice site. Only when such positive identification of splicing signals has occurred will the rest of the splicing machinery be recruited to the pre-mRNA.

We quantified an increased accumulation of spliceosomal components on the pre-mRNA that was dependent on the number of introns within the gene sequence. These observations assist in understanding the dynamics of spliceosome assembly on multiple introns and would be consistent with the scenario of a step-wise model of spliceosome assembly on each intron [65], as well as exon-tethering by the polymerase [66]. Our live-cell analysis suggests that the polymerase does not wait for splicing to take place but rather runs ahead such that more introns accumulate. Together with the exon-tethering model it would mean that exons are being held through the CTD and thereby recruiting the spliceosome for each splice site.

The kinetic approach we used to measure Pol II transcription kinetics enables the quantification of elongation that is free of influences of initiation at the promoter [34]. The region in which elongation is measured is the MS2 portion of the mRNA in which the generation of each additional MS2 stem-loop results in an increase of YFP-MS2 signals on the transcription site. We inserted the MS2 region downstream of the exon-intron-exon modules and at the 3′-end of the mRNA, thereby monitoring the elongating polymerase when associated with the splicing machinery. When we compared the elongation kinetics of the polymerase moving through the MS2 region after transcribing an E3 or E4 mRNA, we found that the kinetics were similar and in fact did not differ than when transcribing an intronless E1 mRNA. A recent study examining transcription of a variety of endogenous genes along intronic and exonic regions using quantitative RT-PCR has shown that polymerase rates were similar on all genes and did not differ when transcribing intron or exon regions [21].

By generating a gene with many and closely spaced introns and exons (E6), we created a situation in which the transcription machinery should have reached the end of the gene before full splicing of the transcript was completed. Although the generation of the E6 and E4 genes required the duplication of intron 2 and the flanking splice sites, we did not find that this had an effect on the splicing outcome (RT-PCR) of E6 and E4 pre-mRNAs, nor on the expression levels of the CFP-peroxisome protein product. Also, there was no indication of aberrant localization of the E6 mRNA on the transcription site or in nuclear speckles, which could be indicative of aberrant mRNA production.

The FRAP recovery kinetics of the E6 gene were significantly slower than the E3 and E4 genes. In order to examine whether the polymerase was the cause of these slow kinetics, or if the splicing machinery was responsible, we conducted a series of experiments. Using splicing inhibitors we saw that the impediment of the slow kinetics was released and returned to the levels of E3/E4/E1 genes, demonstrating that the slow-down was due to an active splicing environment. On the other hand, GFP-Pol II recovery kinetics and ChIP distribution patterns demonstrated that the polymerase was not paused or hindered during elongation. Use of actinomycin D that inhibits transcription by intercalating into the DNA and obstructing the path of the polymerase demonstrated that the clearance of the mRNAs from the E6 gene was slower. These transcripts were polyadenylated as seen by recruitment of PABP2 and the presence of polyA tails. The four different transcripts showed polyA accumulation on the transcription sites implying that the transcripts remained attached to the gene until 3′-end processing occurred. The quantification of the ratio of polymerase to nascent transcripts showed that there was accumulation of pre-mRNAs on the E6 gene, but not on the other genes. This accumulation was alleviated when splicing was inhibited. Together with parameter estimation by the simulation we demonstrate that unspliced E6 transcripts are retained at the site of transcription for up to 10 min before being released.

The plausibility of a co-transcriptional splicing mechanism can be imagined just by the fact that human gene exons tend to be short compared to extremely larger intronic sequences [67], and genes contain an average of 5–12 exons [68]. This means that if co-transcriptional splicing did not take place, then the splicing machinery would have to hold back until transcription has completed. This would not make much biological sense, especially when envisaging the transcriptional timeframe of very large genes such as the dystrophin gene, which lies in the range of 16 h [69]. In fact it has been proposed that the immediate association of U1 snRNP and splicing factors with the emerging pre-mRNA is a means to block the association with hnRNPs, which are splicing inhibitory proteins [46]. We demonstrated that the polymerase activity is uncoupled from splicing once transcription has terminated. In a scenario in which transcription has ended and splicing continues, the pre-mRNA and splicing machinery are retained at the site of transcription until the completion of splicing.

## Materials and Methods

### Plasmid Construction

E4 generation: The E3 construct (termed E3-minus in [37]) was digested with BstXI. Re-circularization was prevented by Shrimp Alkaline Phosphatase (Fermentas). An adaptor composed of the hybridized A1 & A2 sequences was ligated using T4 DNA ligase (5u/µl, Fermentas, Ontario, Canada). A1:TTGATATCACCGGTGGGCCCGGAAAAGAATTCGTCCATCACT

A2:ATGGACGAATTCTTTTCCGGGCCCACCGGTGATATCAAAGTG

The adaptor was EcoRV/BstXI digested, and a PmlI/BstXI intron2-exon3 insert that was separately removed from E3 was then ligated into the adaptor to generate E4.

E6 was constructed using the same strategy but on the E4 construct. E1 was generated by first digesting the E3 plasmid BstXI/SacII, followed by ligation with an adaptor composed of A9 & A10.

A9:AAGCTTTGATCAGCGGCCGCATCACT

A10:GGTTCGAAACTAGTCGCCGGCGTA

RNA extractions were carried out with the RNeasy mini kit (Qiagen). cDNA was synthesized using the RevertAid™ First Strand cDNA Synthesis Kit and an oligo(dT) primer (Fermentas). RT-PCR and sequencing of the correctly spliced mRNAs were performed with primers to the first exon and last exon regions.

A3: AGCAACCTCAAACAGACACC

A4: GGTCTTGTAGTTGCCGTCGT

Other plasmids used in this study were: RFP-LacI, CFP-LacI, YFP-MS2(-NLS), GFP-Pol II [34], (NES-)YFP-MS2(-NLS) [36], MS2-mCherry, GFP-PABP2, GFP-PTB, GFP-TLS, and GFP-cyclin T1.

### Cell Culture and Transfections

Human U2OS osteosarcoma Tet-On cells (Clontech) were maintained in low glucose Dulbecco's modified Eagle's medium (DMEM, Biological Industries, Israel) containing 10% fetal bovine serum (FBS, HyClone, Logan, UT). For transient transfections, cells were transfected with 1–5 µg of plasmid DNA and 40 µg of sheared salmon sperm DNA (Sigma) using electroporation (Gene Pulser Xcell, Bio-Rad, Hercules, CA) or with FuGENE 6 transfection reagent (Roche). Stable co-transfections were performed by electroporation of the E genes together with a 256 *lacO* repeat plasmid and a puromycin resistance plasmid using a 1∶10∶10 ratio (puromycin res., *lacO*, E gene). Cells were cultured for 1–2 d without selection and then selected for 2–3 wk with 100 µg/ml puromycin (AG scientific, San Diego, CA). Single colonies were picked and positive clones were selected after the addition of doxycycline (1 µg/ml) and identification of CFP-peroxisomes and by transient transfection of RFP-LacI and YFP-MS2. For drug treatments cells were imaged before and right after the addition of 5 mg/ml actinomycin D (Sigma). For DRB treatment, cells were treated with 20 µg/µl for 2 h before imaging. For splicing inhibition, cells were photobleached before and after incubation with 100 ng/ml of Spliceostation for 6 h, kindly provided by Minoru Yoshida (RIKEN Advanced Science Institute, Japan), or Meayamycin (300 nM) for 6 h before imaging, kindly provided by Kazunori Koide (University of Pittsburgh, USA).

### qPCR

gDNA was isolated using the PUREGENE™ DNA Purification System (Gentra). For the generation of standards a 10-fold dilution was prepared. Primers used for the amplification of CFP region were GGATCACTCTCGGCATGGAC (forward) and TGCACATACCGGAGCCATTG (reverse). Primers used for the human β-globin reference gene were CAGTGCAGGCTGCCTATCAGA (forward) and GAATCCAGATGCTCAAGGCCCTT (reverse). The reaction (20 µl total volume) contained 1× SYBR green mix (Thermo scientific, Waltham, MA), 1 µl of gDNA dilution, and 0.5 mM of each primer and water. The reaction was performed using the Chromo4™ Real-Time Detector (Biorad) following the protocol: 15 min at 95°C for enzyme activation, followed by 40 cycles consisting of 15 s denaturation at 92°C, 10 s annealing at 64°C, and 20 s extension at 72°C. Fluorescent signal was measured at the end of each cycle. A final dissociation stage was performed to generate a melting curve for verification of amplification product specificity. For calculating the gene copy number, we first calculated the reaction efficiency for each set of primers by using triplicates of 5× dilutions of gDNA, using the provided software Opticon2 (Biorad). We measured the average cycle number for each gDNA sample using both primer sets and calculated the concentration (Qc) of each reaction by: 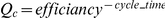. Then we could calculate the gene copy number of the samples: 
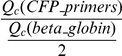


We verified that the U2OS cell line has only two alleles of the *β-globin* gene by comparing to a non-cancerous cell line.

### Chromatin Immunoprecipitation and Quantitative PCR

ChIP and quantitative PCR were performed as previously described [18]. After an overnight induction with 1 µg/ml doxycycline, cells were harvested and lysed in SDS lysis buffer. Protein concentration was determined via Amido Black and 1 mg total protein was used per IP. For SSA treatment, cells were treated with 100 ng/ml SSA prior to lysis. Antibodies used for the ChIP were 5 µg/IP CTD4H8 against RNA polymerase II (Santa Cruz Biotechnology, SC-47701) and 10 µg/IP non-immune mouse IgG (Sigma, I5381) for the mock ChIPs. Uncrosslinking and Proteinase K treatment (100 µg/IP) were performed for 6 h at 65°C. DNA was extracted using the Qiagen PCR purification kit and used as a template for quantitative PCR. Primer sequences used in the qPCR analysis can be obtained upon request. For each amplicon, ChIP signal relative to input was calculated based on the Ct values. The values obtained for control antibody were then subtracted from those of specific antibodies. Thus, the following equation was used: 2(Ct(Input)−Ct(spIP))−2(Ct(Input)−Ct(cIP)), where: spIP−IP with specific antibody and cIP−IP with a control antibody. Values obtained for amplicons within gene regions were further normalized to the promotor region.

### Immunofluorescence

Cells grown on coverslips were fixed for 20 min in 4% PFA and then permeabilized in 0.5% Triton X-100 for 3 min. After blocking with 5% BSA, cells were immunostained for 1 h with: mouse anti-RNA Pol II antibody (clones H14, H5, 8WG16 (Covance)), mouse anti-SF2/ASF (Zymed), mouse anti-PSF (B92, Sigma), mouse anti-2,2,7-trimethylguanosine (TMG) (Mercury), rabbit anti-U1A, rabbit anti-U2AF65 (Abcam), mouse anti-SC35 (Sigma), mouse anti-9G8, and mouse anti-p54^nrb^ (BD Bioscience). After subsequent washes the cells were incubated for 1 h with a secondary Ab: Cy5-labeled goat anti-mouse IgG, Cy3-labeled goat anti-mouse IgG, FITC-labeled anti-mouse IgM (Jackson ImmunoResearch, West Grove, PA), Alexa488-labeled goat anti-mouse IgG, Mouse anti-rabbit Alexa488, and rabbit anti-mouse Alexa594 (Molecular Probes). Coverslips were mounted in mounting medium.

### Fluorescence In Situ Hybridization

RNA FISH was performed on dox activated cells as previously described [70] using 10 ng (probe per slide) of 5′-Cy3 or Cy5 labeled fluorescent probes, as follows:

CFP: ATATAGACGTTGTGGCTGATGTAGTTGTACTCCAGCTTGTGCCCCA

GGATA

Exon1: CATGAATTCTTTGCCAAAGTGATGGGCCAGCACACAGACCAGCA

CGTTGC

Intron1: TCTCGTGACAGAGAAGGGTGTAAAAGCTTCTAGCCTTTTCTCTTA

CCTTA.

Intron2: TAGCAAAAGGGCCTAGCTTGGACTCAGAATAATCCAGCCTTATC

CCAACC

MS2: CTAGGCAATTAGGTACCTTAGGATCTAATGAACCCGGGAATACT

GCAGAC

PolyA: an oligo(dT) probe

The U snRNA set of probes containing 5-amino-allyl thymidines (Eurogentec) were labeled with Cy3 or Cy5 monoreactive dyes (GE Healthcare).

U1-1: CtGGGAAAACCACCtTCGTGATCAtGGTATCTCCCCtGCCAGGTAAGtAT

U1-2: CGAACGCAGtCCCCCACtACCACAAATTAtGCAGTCGAGtTTCCCACAtT

U2: AGtGGACGGAGCAAGCtCCTATTCCAtCTCCCTGCtCCAAAAATCCATtT

U4-1: tAGCAATAAtCGCGCCTCGGAtAAACCTCATtGGCTACGATACtGCCACT

U4-2: AGACtGTCAAAAATtGCCAATGCCGACtATATTGCAAGtCGTCACGGCGGtA

U5: GGCAAGGCTCAAAAAATTGGGTTAAGACTCAGAGTTGTTCCTCTCCACGGTA

U6: CGtGTCATCCTtGCGCAGGGGCCAtGCTAATCTTCtCTGTATCGTtCCAA

7SK snRNA: TtGGATGTGTCTGGAGTCTtGGAAGCTTGACTACtT

In some cases, an immunostaining was performed after FISH using the standard protocol.

### Fluorescence Microscopy, Live-Cell Imaging, and Data Analysis

Wide-field fluorescence images were obtained using the Cell∧R system based on an Olympus IX81 fully motorized inverted microscope (63× Plan-Apo, 1.42NA and 100× Plan-Apo, 1.4NA) fitted with an Orca-AG CCD camera (Hamamatsu), rapid wavelength switching, active focus control (ZDC), a motorized stage (Scan IM, Märzhäuser, Wetzlar-Steindorf, Germany), and driven by the Cell∧R software. The microscope is equipped with an on-scope incubator which includes temperature and CO_2_ control (Life Imaging Services, Reinach, Switzerland).

For time-lapse imaging of ActD treatment, several cell positions under non-treated conditions were chosen and recorded. Then 5 mg/ml of actinomycin D was added and cells were imaged every 5 min for 1.5 h.

Images and videos were analyzed using self-written ImageJ software macros (National Institutes of Health, Bethesda, MD). The co-localization intensity profile was generated with an ImageJ macro. The channel intensity correlation was generated by Matlab. The 4D image sequences were sum Z-projections. The transcription site was tracked and the mean intensity was measured at every time-point by finding the local maxima of the red (RFP-LacI) for each time point in a specific region of interest (ROI). The background was taken from an ROI outside of the cell and subtracted from all other measurements. Photobleach correction was obtained using another area in the nucleus or in another nucleus. Images acquired before drug addition were used as the initial conditions for normalization of the data. The half-time of site inactivation was calculated for each transcription site using Matlab. Each transcription site measured intensity dataset was fitted to a bi-exponential equation f(x) = a*exp(b*x)+c*exp(d*x). We then searched for the 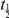, which brings the equation to the value of 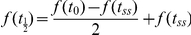. For each E cell type we collected at least 7 cell transcription site measurements, and the averages and standard error were calculated.

### FRAP

FRAP experiments were performed using a 3D-FRAP system (Photometrics) built on an Olympus IX81 microscope (63× Plan-Apo, 1.4 NA) equipped with an EM-CCD (Quant-EM, Roper), 405 & 491nm lasers, Lambda DG-4 light source (Sutter), XY&Z stages (Prior), and driven by MetaMorph (Molecular Devices). Experiments were performed at 37°C with 5% CO_2_ using a live-cell chamber system (Tokai). Before and after the bleaching, cells were imaged in the YFP channel for the detection of YFP-MS2 (mRNPs) and in the CFP channel for the detection of CFP-lacI (genomic locus). For each acquisition, 7 Z-slices were taken every 350 nm. The active transcription site was bleached using the 491 nm laser. Six pre-bleach images were acquired. Post-bleach images were acquired in a sequence of 3 time frequencies: 15 images every 3 s, 15 images every 6 s, and 26 (or 45 for long experiments) images every 30 s.

Experiments were performed on two additional systems to verify their consistency: (1) a similar 3D FRAP system in the Darzacq laboratory under the same conditions; (2) Zeiss LSM 510 Meta laser scanning confocal microscope (Plan-Apochromat 63×, 1.4 NA oil objective (Jena, Germany)). Cells were scanned at 488 nm for GFP-MS2 or 514 nm for YFP-MS2, together with a 543 nm laser for the detection of RFP-lacI at the genomic locus. Post-bleach recovery images were acquired every 1 s.

The experiments were analyzed using self-written ImageJ macros that sum the projected Z-stacks at every time-point, and track and measure the mean intensity of the transcription site at every time-point by finding the local maxima in a specific region of interest (ROI). For each time-point, the background taken from a ROI outside of the cell was subtracted from all other measurements. T(t) and I(t) were measured for each time-point as the average intensity of the nucleus and the average intensity in the bleached ROI, respectively. The average of the pre-bleach images used as the initial conditions are referred to as Ti  =  nuclear intensity and Ii  =  intensity in ROI before bleaching. Ic(t) is the corrected intensity of the bleached ROI at time t [42],[71]: 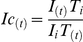


Data from at least 10 experiments for each cell-line were collected and the averaged FRAP measurements were fitted to the simulation.

The diffusion of the YFP-MS2 protein during the FRAP analysis was disregarded since the diffusion rate of free nucleoplasmic YFP-MS2 is very rapid, while the bound YFP-MS2 is associated with high affinity to the mRNA, and does not detach, diffuse, and bind again at the transcription site [34],[41],[42].

### Quantitative RNA FISH and Immunofluorescence Analysis

Quantitative RNA FISH was used to compare the ratio of FISH signals from two different probes in two different channels. Although each cell line had a different number of gene-copies integrated, the ratio per transcript is comparable. Z stacks (91 slices, 200 nm steps) of the immunostained or RNA FISH samples were collected. Images were acquired for the quantification of the exons, introns, U snRNAs, or Pol II present on the transcription sites. Each experiment in which all E cell types were analyzed were acquired under the same conditions and on the same day. Images were deconvolved using a point spread function (PSF) based deconvolution algorithm Huygens Essential II (Scientific Volume Imaging, Hilversum, The Netherlands). The sum of pixel values at the transcription site in each channel were measured using Imaris (Bitplane, Saint Paul, MN), and the ratio between the channels is presented.

### Computational Simulation

Monte Carlo simulations (Matlab) of the transcriptional process were based on the mechanistic models of [34],[41]. The simulation was used to simulate the FRAP experiments, and by fitting the simulation to the experimental data we could retrieve the kinetic parameters of the process. The simulation performs stochastic decisions by using random numbers obtained from the simulation and checks whether they are smaller than the kinetic parameters. For each gene (E1, E3, E4, and E6), we generated a set of identical arrays where the length of each array corresponded to the length of the gene. RNA polymerases were implemented as counters that “slide” over these arrays. “Transcriptional initiation” occurred stochastically at a given “initiation rate,” after which a constant speed of elongation was retained. Whenever a polymerase moves through the “MS2 region” of the gene, the polymerase/transcript accumulates a “fluorescent signal” that was maintained until the end of the gene. Polymerases were stochastically released at a given termination rate [41]. Polymerases could also randomly enter and exit a paused state [34]. The simulation reached steady state after long times, as expected.

To simulate the FRAP experiments, we tracked the total “fluorescent” signal. After the system entered steady state, we “bleached” (deleted) the fluorescent molecules and measured the buildup of “fluorescent” signal during the recovery. The signal was normalized as in the FRAP experimental procedure. The kinetic parameters, namely, the elongation speed, the termination rate, and the rates of entering and exiting the paused state, were chosen as those that best fit the experimental FRAP data. We found that the number of gene arrays and the initiation rate had no effect on the measured kinetic results, due to the normalization to the pre-bleach signal.

To find the above four kinetic parameters that best fit the experimental data we had to explore a four-dimensional parameter space. For each configuration, the mean square difference (MSD) between the simulated FRAP curve and the experimental curve was calculated (Figure S10). For the E3 gene, the kinetic parameters that gave the best fit (lowest MSD) were: 3.6 kb/min, ∼42% of the polymerases entering a paused state for an average of 6 min, and transcript retention of 50 s after the end of elongation. To obtain the kinetic parameters for the E6 gene, we used the best fit values of the E3 elongation speed and pausing transition rates, and changed only the termination rate.

## Supporting Information

Figure S1 **Transcriptional induction of the different cell lines.** The cell lines containing the stably integrated E1, E3, E4, and E6 genes were transcriptionally induced by dox for 12 h. RNA-FISH with a probe to the MS2 region shows the transcription sites (yellow). The CFP protein product is targeted to peroxisomes (cyan). DIC on the right (bar, 5 µm).
(3.64 MB TIF)

Figure S2 **Recruitment of RNA processing factors to active transcription sites of E3 cells.** The gene locus was identified with RFP-LacI (red) and the recruitment of endogenous Pol II (green) was identified by (A) 8WG16 Ab (specific to the CTD repeats) and (B) H5 Ab (CTD phosphorylated on serine 2). Active transcription sites (MS2, yellow) can be seen together with immunofluorescence (red) with (C) anti-TMG identifying the cap structure of snRNAs. (D) RNA-FISH to U4 snRNA and (E) U5 snRNA, together with a probe to the MS2 region (yellow). (F) Immunofluorescence with anti-U1A, (G) anti-U2AF65, (H) anti-SF2, (I) anti-9G8, and (J) anti-SC-35; together with the YFP-MS2 protein. Enlargements show the merged signals at the transcription sites. Plots depict the degree of co-localization of the signals at the transcription site across the depicted line (bar, 5 µm).
(3.54 MB TIF)

Figure S3 **Recruitment of transcription-related factors to active transcription sites of E3 cells.** (A) Transiently expressed GFP-TLS, (B) GFP-PSF, (C) GFP-p54^nrb^, (D) GFP-PTB, (E) GFP cyclin T1 (pseudocolored red), imaged together with YFP-MS2. (F) RNA-FISH to 7SK RNA (red) and MS2 repeats (yellow). Enlargements show the merged signals at the transcription sites. Plots depict the degree of colocalization of the signals at the transcription site across the depicted line (bar, 5 µm).
(3.78 MB TIF)

Figure S4 **Recruitment of RNA processing factors to transcription sites of the intronless E1 gene.** Immunofluorescence (red) with (A) anti-TMG that identifies the cap of snRNAs, (B) anti-U1A, and (C) anti-U2AF65, together with the mRNA seen by transfected YFP-MS2 labeling (yellow). (D) RNA-FISH to U4 snRNA and (E) U5 snRNA, together with a probe to the MS2 region (yellow). Enlargements show the signals at the transcription sites. Plots depict the degree of co-localization of the signals at the transcription site across the depicted line (bar, 5 µm).
(1.67 MB TIF)

Figure S5 **Comparison of FRAP results on E3 cells performed on different microscopes.** The recovery of photobleached YFP-MS2 on actively transcribing sites was monitored in E3 cells using: (a) a Zeiss confocal microscope; (b) a 3D FRAP system in our laboratory (system 1); and (c) a 3D FRAP system in the Darzacq laboratory (system 2) (average from at least *n*>10 in each experiment).
(0.14 MB TIF)

Figure S6 **Genomic positioning and gene copy number have no effect on the measured kinetics.** (A) FRAP results of different E6 clones (E6-3 and E6-22-22) performed on different microscopes. (B) Real-time PCR on genomic DNA was used to quantify the number of genes integrated into each gene array gene repeats: E3  =  24, E4  =  5, E6-22-22  =  40, E6-3  =  9 with standard deviation.
(0.46 MB TIF)

Figure S7 **Analysis of FRAP curves. FRAP recovery curves of the E3 transcription site show bi-phasic kinetics, which points to two parallel processes.** The red line is the curve fit for the sum of the two-exponential equation and shows a better fit than the green line that was fitted to one exponential. The yellow line presents the fast phase (70%) and the pink line is the slow phase (30%).
(0.15 MB TIF)

Figure S8 **The effects of splicing inhibitors on the elongation kinetics.** (A) Inhibition of splicing by Meayaymycin (6 h) showed that the unspliced pre-mRNA was distributed throughout the cell and in speckles (RNA-FISH on E6 cells) and was retained in the nucleus (bottom), whereas in untreated cells pre-mRNA was detected only at the site of transcription. (B) FRAP recovery curves of untreated and Meayamycin-treated E6 cells shows similar kinetics as E3 cells. (C) The treatment of cells with SSA for 9 h followed by FRAP analysis of GFP-Pol II recruited to the transcription sites shows that splicing inhibition did not affect polymerase kinetics.
(1.51 MB TIF)

Figure S9 **Searching the kinetic parameter space for the best fit to experimental data.** (A) Simulation plots of an elongating (red) or paused (blue) polymerase population. Top part shows an example of an experiment in which the MS2 fluorescence accumulates (arrow) and is then released. Bottom part shows the accumulation of fluorescence in the MS2 region (left arrow) as well as a stochastically paused polymerase (right arrow). (B,C) The simulations rely on four kinetic parameters: the elongation speed, the termination time, and entering and exiting rates of the paused state (K_in_, K_out_). To find the best fit to the experimental data, the kinetic parameters were varied in a systematic way. The parameters were plotted two at a time because the parameter space is four dimensional. (A) Plot of the mean square difference (MSD) between the experimental FRAP curve and the simulated curve for each pair of elongation speed and termination time. (B) Similar analysis with the pausing transition rates. The MSD is color coded: the dark blue corresponds to parameter combinations that result in the best fit to the experimental data.
(1.25 MB TIF)

Figure S10 **Exploring how the simulation parameters affect the kinetics.** (A) Changes in the elongation speed. (B) Changes in the probability to enter or exit a paused state changed the pausing time during transcription, and the average percentage of polymerases that paused in steady state. (C) Changing only the termination time.
(0.64 MB TIF)

Figure S11 **Low DRB does not affect kinetics on E3 and E6 genes.** No change in the FRAP recovery kinetics of YFP-MS2 on cells treated with DRB (20 µg/µl for 2 h before imaging): E3 cells (brown), E6 cells (dark green), or E6 cells with SSA (cyan) SSA; compared to untreated E3 (blue) and E6 (light green) cells.
(0.14 MB TIF)

Video S1 **Transcriptional activation of E3 (top) and E6 (bottom) cells.** The DNA locus is tagged with CFP-LacI and the cytoplasmic peroxisomes are also seen in CFP. The mRNA is tagged with YFP-MS2. As transcriptional induction proceeds, the transcription sites appear and then mRNAs disperse into the nucleoplasm. CFP-peroxisomes appear at later times. Since the videos were acquired in 3D and the z-stacks covered the cell volumes, we could show the nuclear CFP-LacI locus in the beginning of the videos and then later shift to the cytoplasmic plane that best shows the CFP-peroxisomes. Cells were imaged 15 min after dox addition and the time in the videos is measured from dox addition. E3, imaged every 15 min; E6, imaged every 20 min.
(1.59 MB MOV)

Video S2 **FRAP experiment of an E4 cell. mRNA was labeled with YFP-MS2.** 2D image over time of a summed projection of a 4D video. Right: Enlarged tracking of the transcription site. Time zero indicates the bleach time.
(0.28 MB MOV)

Video S3 **Addition of actinomycin D to transcribing E6 cells and recording transcription site inactivation over time.** The mRNA was tagged with YFP-MS2 (green) and the transcription site locus with RFP-LacI (red). Time zero indicates ActD addition time.
(0.94 MB MOV)

Video S4 **Example of a simulation run.** Each dot denotes a polymerase moving along the gene: elongating  =  red dot, and stochastically pausing  =  dot becomes green. When the polymerase terminates and the transcript still remains on the transcription site, the dot becomes yellow. The bleached polymerase becomes blue. The *x*-axis  =  gene length of E3; in the *y*-axis each line represents a gene within the transcription site. Top: a single gene view. Bottom: an array of 1,000 genes running in parallel. The last frame contains a graph showing the MS2 intensity measured from the simulation.
(4.30 MB MOV)

## Abbreviations

lacI: lac repressor protein
lacO: lac operator repeats
