# Supplementary figures and images for: The In Vivo Kinetics of RNA Polymerase II Elongation during Co-Transcriptional Splicing
Source: PLoS Biol. 2011 Jan 11;9(1):e1000573. doi: 10.1371/journal.pbio.1000573 (PMC3019111; doi:10.1371/journal.pbio.1000573)

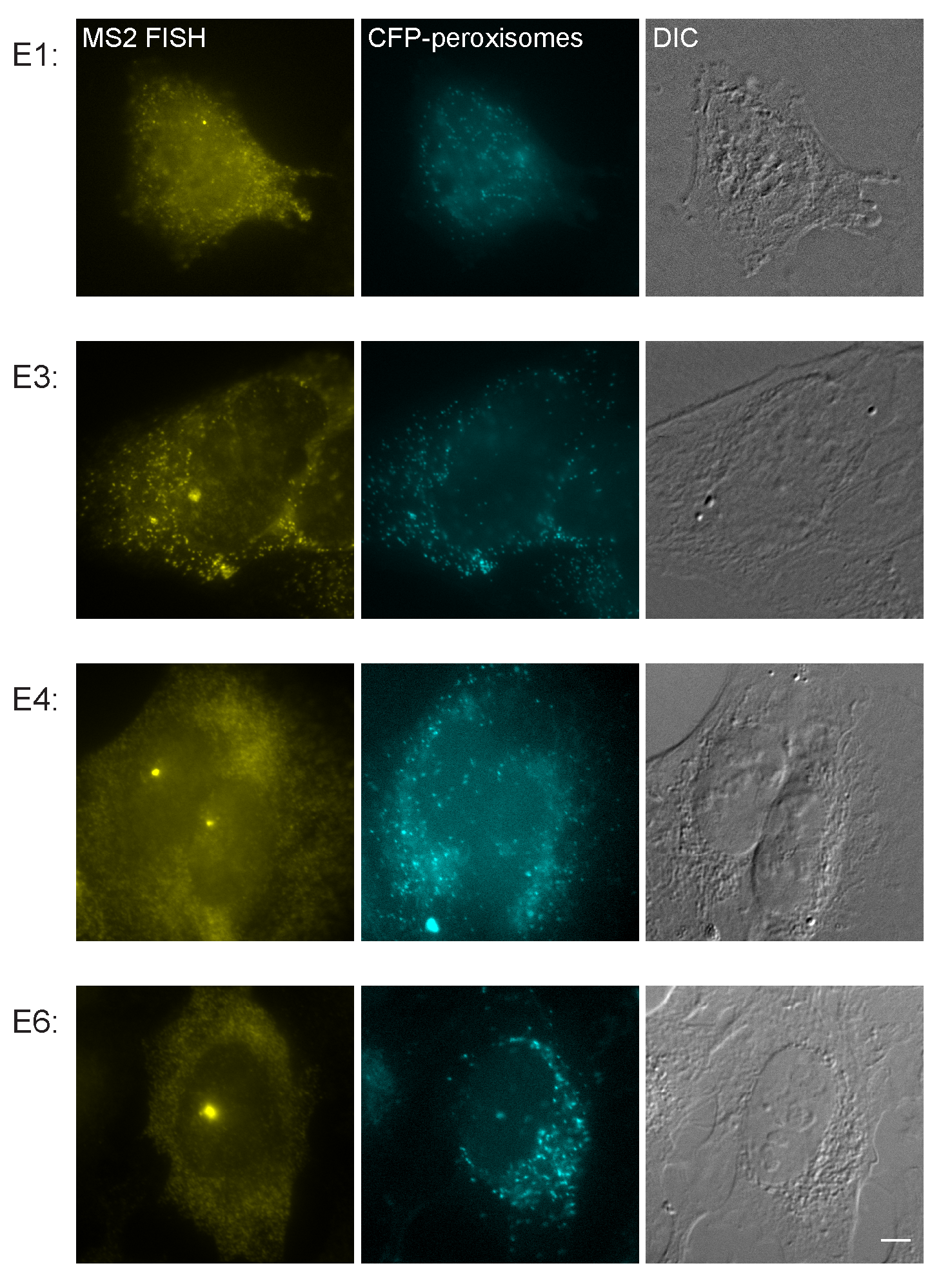

Supplement: Figure S1 — Transcriptional induction of the different cell lines. The cell lines containing the stably integrated E1, E3, E4, and E6 genes were transcriptionally induced by dox for 12 h. RNA-FISH with a probe to the MS2 region shows the transcription sites (yellow). The CFP protein product is targeted to peroxisomes (cyan). DIC on the right (bar, 5 µm). (3.64 MB TIF) [file pbio.1000573.s001.tif]

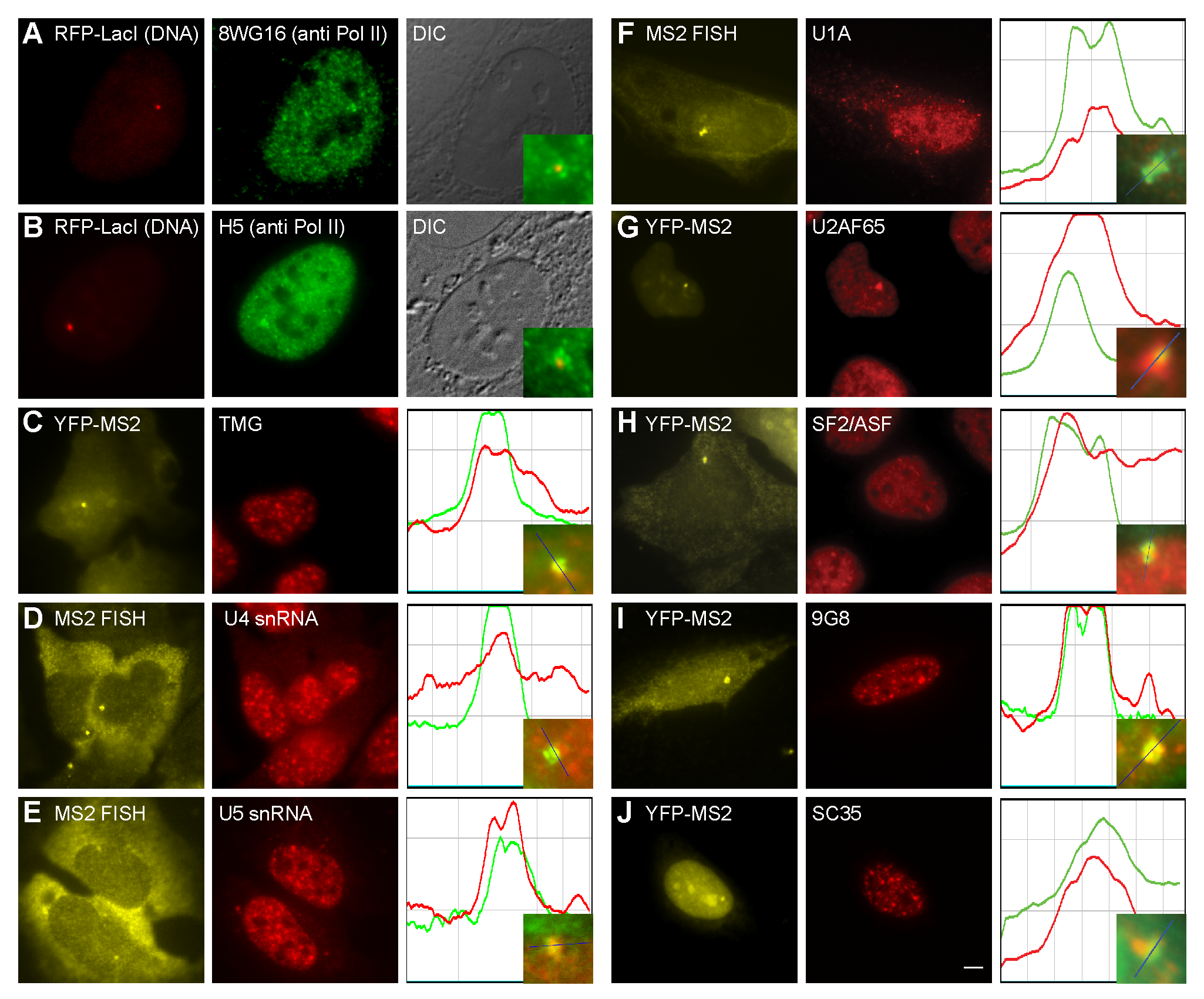

Supplement: Figure S2 — Recruitment of RNA processing factors to active transcription sites of E3 cells. The gene locus was identified with RFP-LacI (red) and the recruitment of endogenous Pol II (green) was identified by (A) 8WG16 Ab (specific to the CTD repeats) and (B) H5 Ab (CTD phosphorylated on serine 2). Active transcription sites (MS2, yellow) can be seen together with immunofluorescence (red) with (C) anti-TMG identifying the cap structure of snRNAs. (D) RNA-FISH to U4 snRNA and (E) U5 snRNA, together with a probe to the MS2 region (yellow). (F) Immunofluorescence with anti-U1A, (G) anti-U2AF65, (H) anti-SF2, (I) anti-9G8, and (J) anti-SC-35; together with the YFP-MS2 protein. Enlargements show the merged signals at the transcription sites. Plots depict the degree of co-localization of the signals at the transcription site across the depicted line (bar, 5 µm). (3.54 MB TIF) [file pbio.1000573.s002.tif]

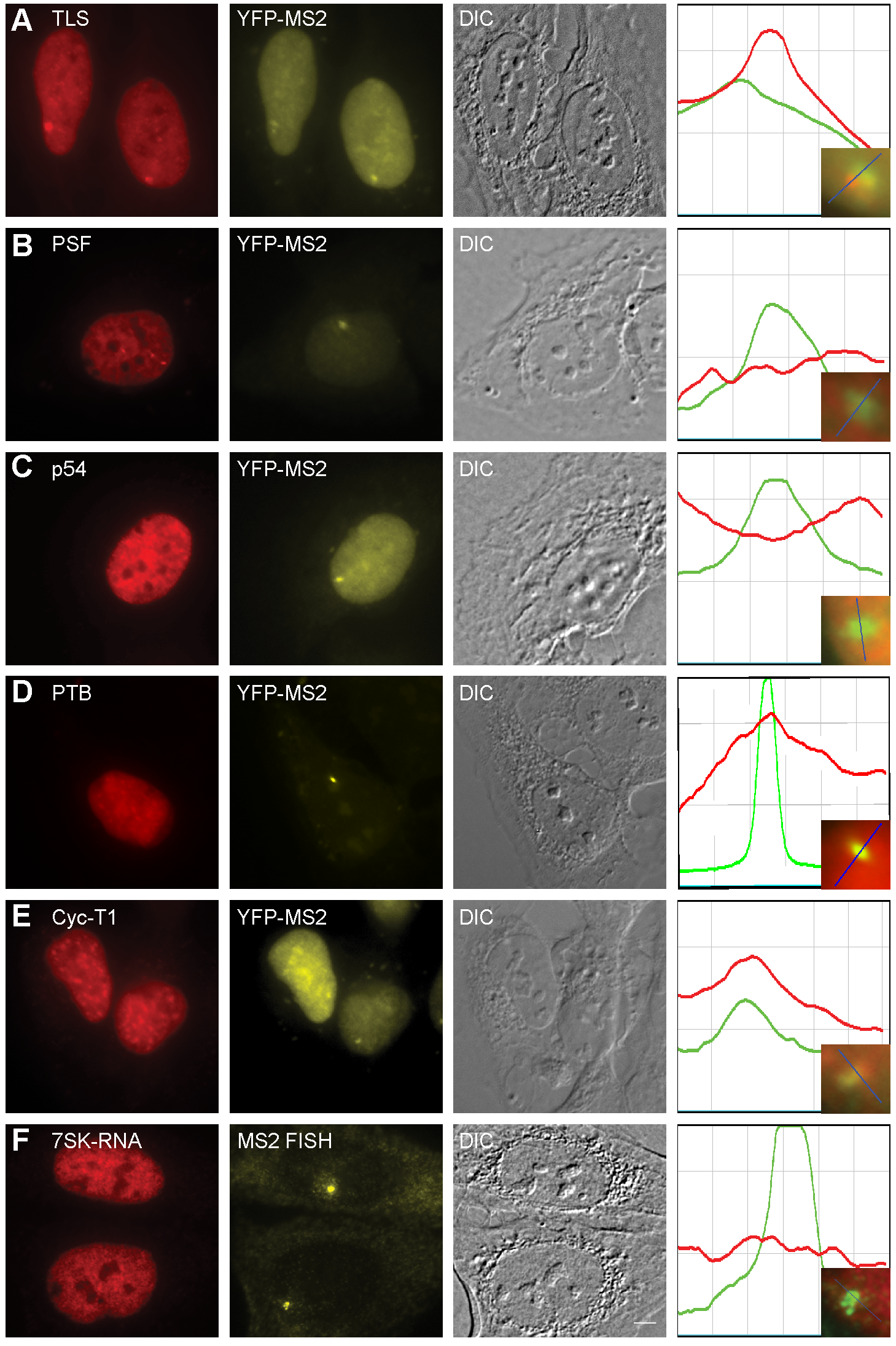

Supplement: Figure S3 — Recruitment of transcription-related factors to active transcription sites of E3 cells. (A) Transiently expressed GFP-TLS, (B) GFP-PSF, (C) GFP-p54nrb, (D) GFP-PTB, (E) GFP cyclin T1 (pseudocolored red), imaged together with YFP-MS2. (F) RNA-FISH to 7SK RNA (red) and MS2 repeats (yellow). Enlargements show the merged signals at the transcription sites. Plots depict the degree of colocalization of the signals at the transcription site across the depicted line (bar, 5 µm). (3.78 MB TIF) [file pbio.1000573.s003.tif]

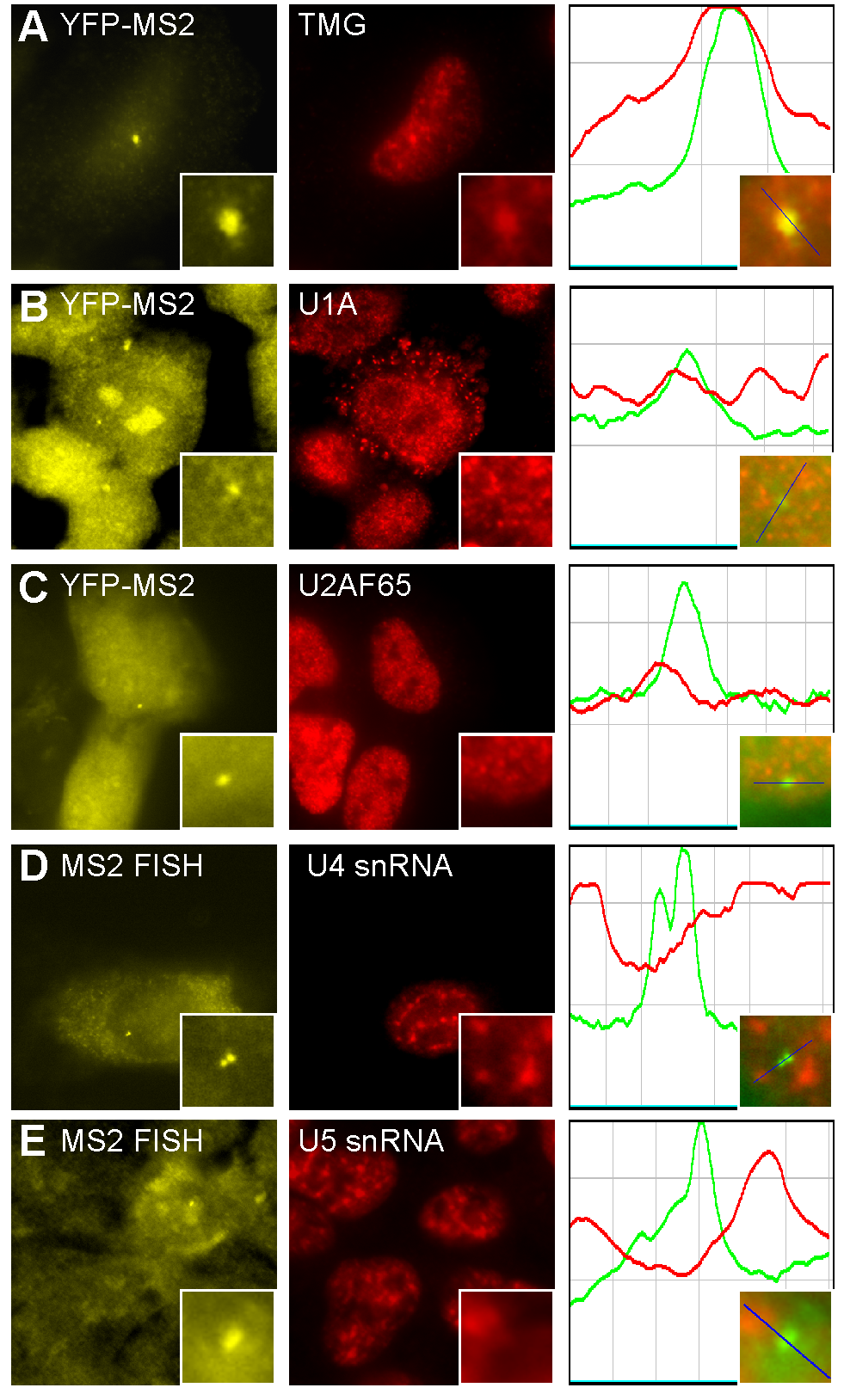

Supplement: Figure S4 — Recruitment of RNA processing factors to transcription sites of the intronless E1 gene. Immunofluorescence (red) with (A) anti-TMG that identifies the cap of snRNAs, (B) anti-U1A, and (C) anti-U2AF65, together with the mRNA seen by transfected YFP-MS2 labeling (yellow). (D) RNA-FISH to U4 snRNA and (E) U5 snRNA, together with a probe to the MS2 region (yellow). Enlargements show the signals at the transcription sites. Plots depict the degree of co-localization of the signals at the transcription site across the depicted line (bar, 5 µm). (1.67 MB TIF) [file pbio.1000573.s004.tif]

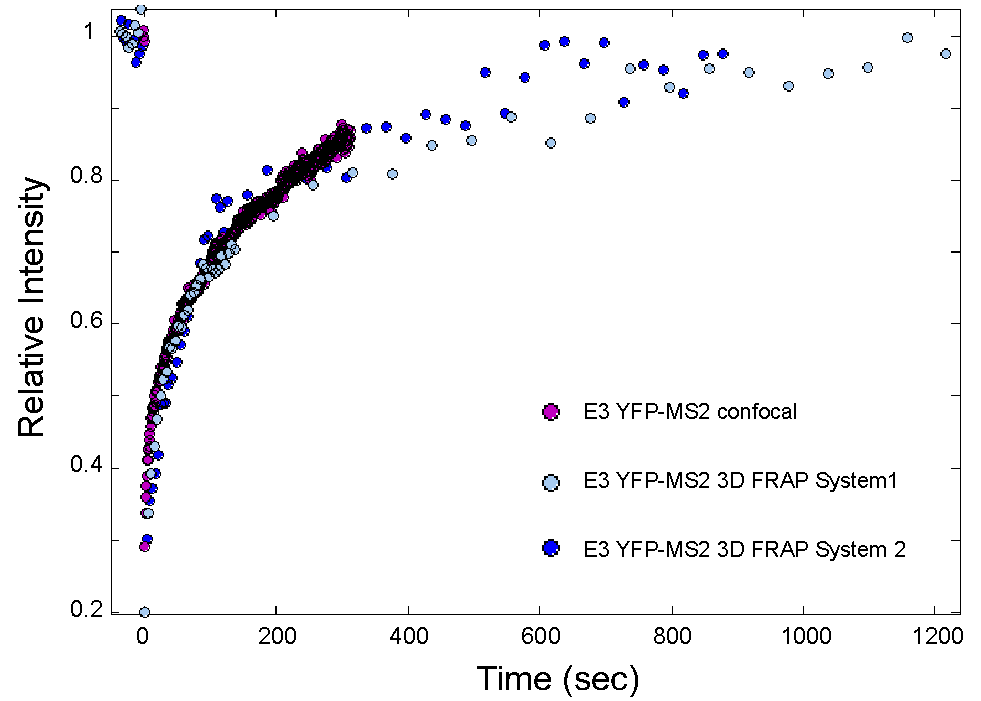

Supplement: Figure S5 — Comparison of FRAP results on E3 cells performed on different microscopes. The recovery of photobleached YFP-MS2 on actively transcribing sites was monitored in E3 cells using: (a) a Zeiss confocal microscope; (b) a 3D FRAP system in our laboratory (system 1); and (c) a 3D FRAP system in the Darzacq laboratory (system 2) (average from at least n>10 in each experiment). (0.14 MB TIF) [file pbio.1000573.s005.tif]

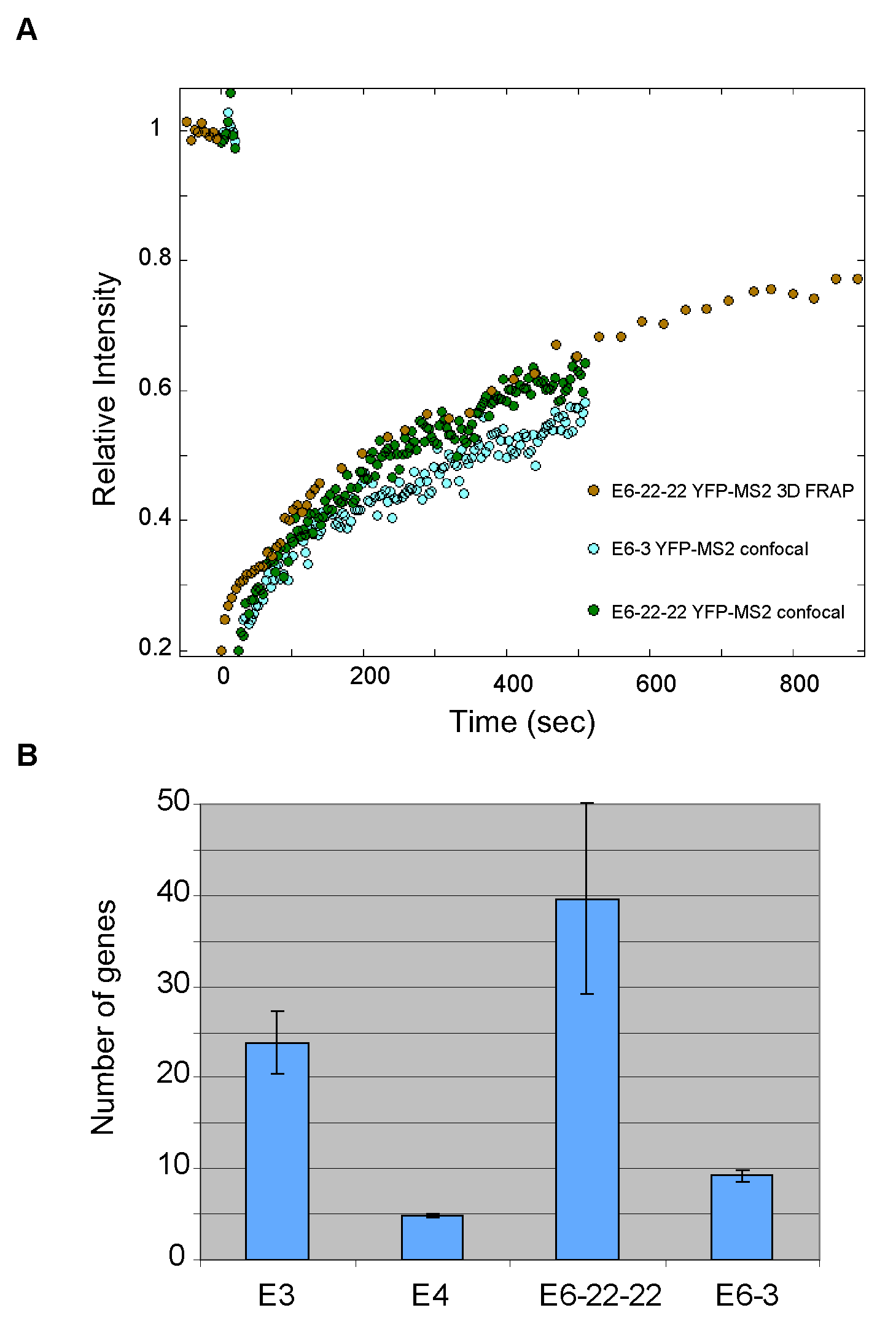

Supplement: Figure S6 — Genomic positioning and gene copy number have no effect on the measured kinetics. (A) FRAP results of different E6 clones (E6-3 and E6-22-22) performed on different microscopes. (B) Real-time PCR on genomic DNA was used to quantify the number of genes integrated into each gene array gene repeats: E3 = 24, E4 = 5, E6-22-22 = 40, E6-3 = 9 with standard deviation. (0.46 MB TIF) [file pbio.1000573.s006.tif]

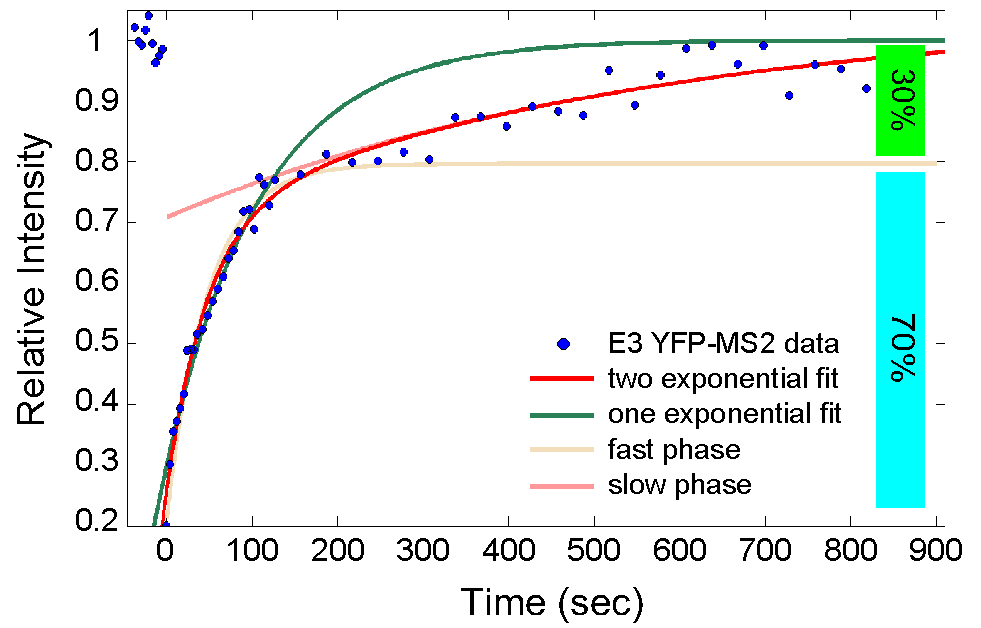

Supplement: Figure S7 — Analysis of FRAP curves. FRAP recovery curves of the E3 transcription site show bi-phasic kinetics, which points to two parallel processes. The red line is the curve fit for the sum of the two-exponential equation and shows a better fit than the green line that was fitted to one exponential. The yellow line presents the fast phase (70%) and the pink line is the slow phase (30%). (0.15 MB TIF) [file pbio.1000573.s007.tif]

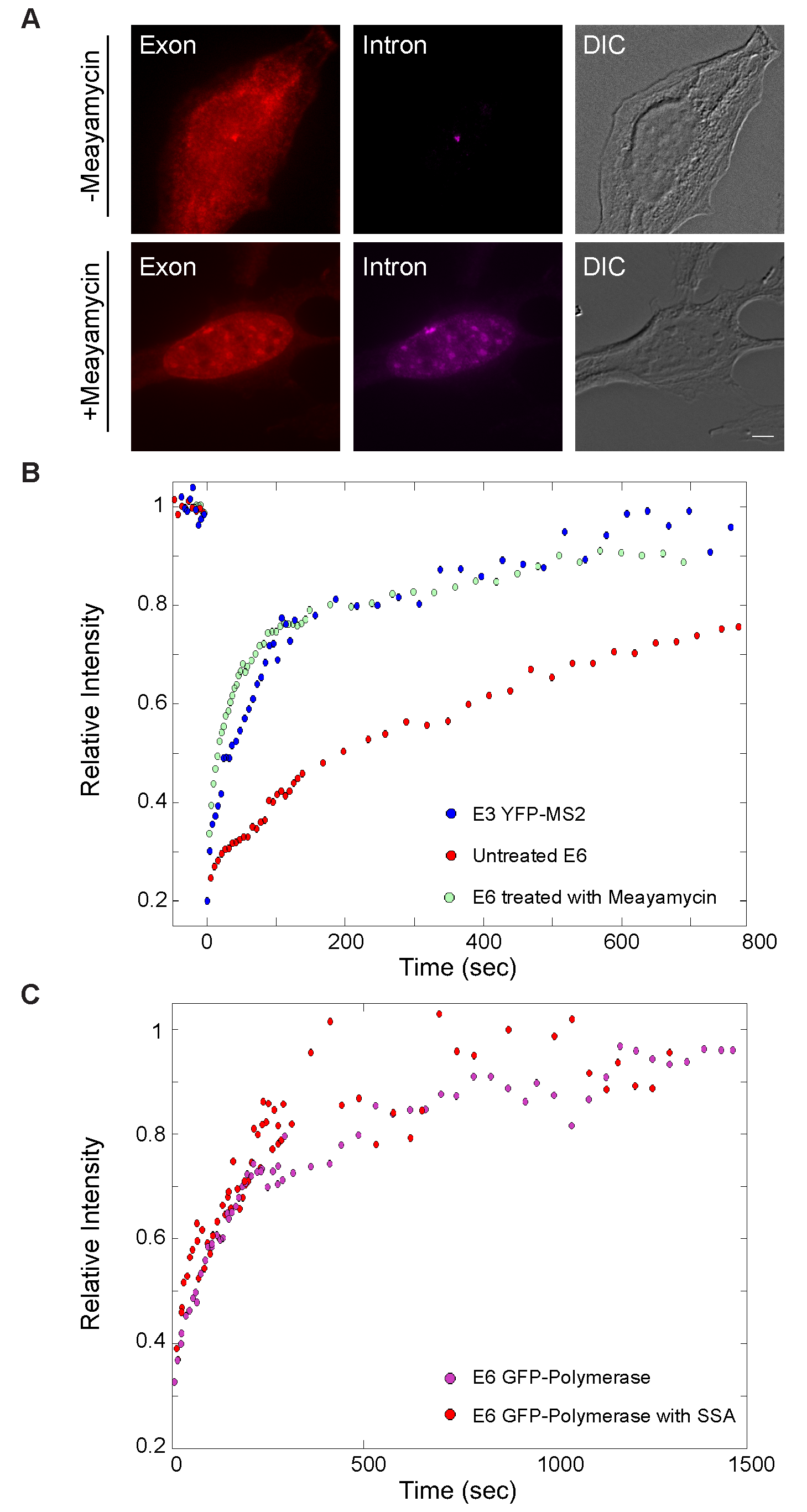

Supplement: Figure S8 — The effects of splicing inhibitors on the elongation kinetics. (A) Inhibition of splicing by Meayaymycin (6 h) showed that the unspliced pre-mRNA was distributed throughout the cell and in speckles (RNA-FISH on E6 cells) and was retained in the nucleus (bottom), whereas in untreated cells pre-mRNA was detected only at the site of transcription. (B) FRAP recovery curves of untreated and Meayamycin-treated E6 cells shows similar kinetics as E3 cells. (C) The treatment of cells with SSA for 9 h followed by FRAP analysis of GFP-Pol II recruited to the transcription sites shows that splicing inhibition did not affect polymerase kinetics. (1.51 MB TIF) [file pbio.1000573.s008.tif]

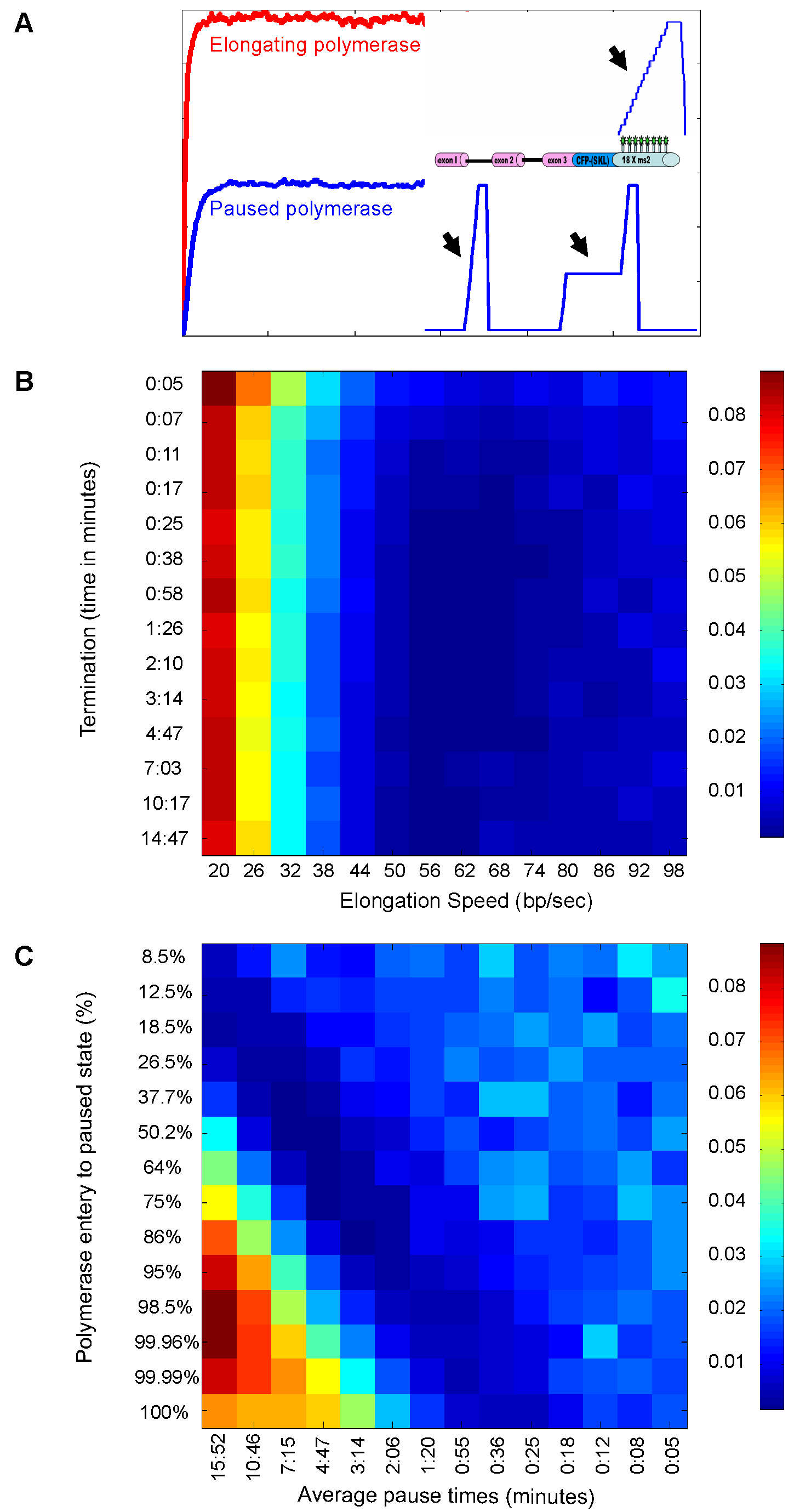

Supplement: Figure S9 — Searching the kinetic parameter space for the best fit to experimental data. (A) Simulation plots of an elongating (red) or paused (blue) polymerase population. Top part shows an example of an experiment in which the MS2 fluorescence accumulates (arrow) and is then released. Bottom part shows the accumulation of fluorescence in the MS2 region (left arrow) as well as a stochastically paused polymerase (right arrow). (B,C) The simulations rely on four kinetic parameters: the elongation speed, the termination time, and entering and exiting rates of the paused state (Kin, Kout). To find the best fit to the experimental data, the kinetic parameters were varied in a systematic way. The parameters were plotted two at a time because the parameter space is four dimensional. (A) Plot of the mean square difference (MSD) between the experimental FRAP curve and the simulated curve for each pair of elongation speed and termination time. (B) Similar analysis with the pausing transition rates. The MSD is color coded: the dark blue corresponds to parameter combinations that result in the best fit to the experimental data. (1.25 MB TIF) [file pbio.1000573.s009.tif]

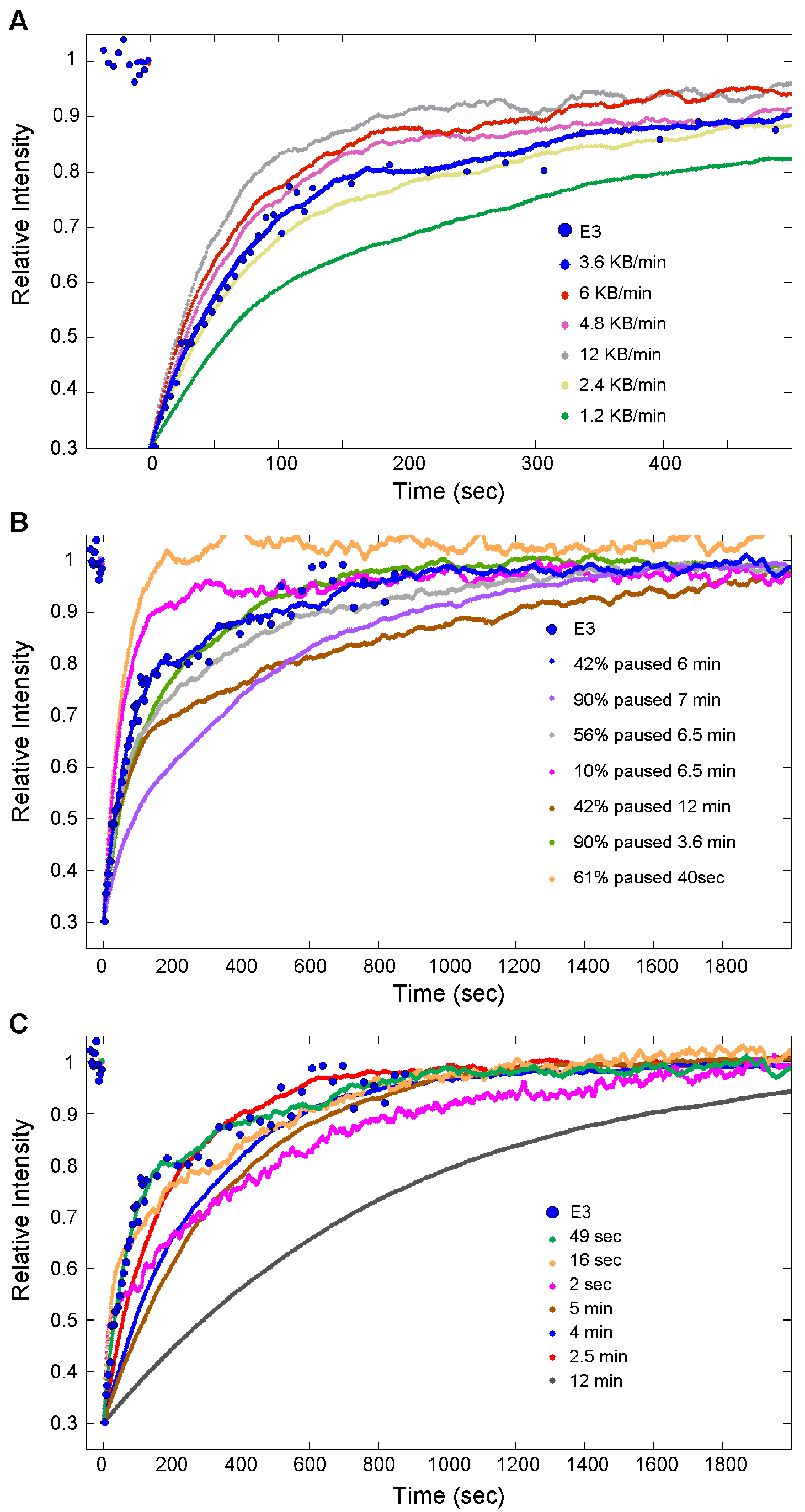

Supplement: Figure S10 — Exploring how the simulation parameters affect the kinetics. (A) Changes in the elongation speed. (B) Changes in the probability to enter or exit a paused state changed the pausing time during transcription, and the average percentage of polymerases that paused in steady state. (C) Changing only the termination time. (0.64 MB TIF) [file pbio.1000573.s010.tif]

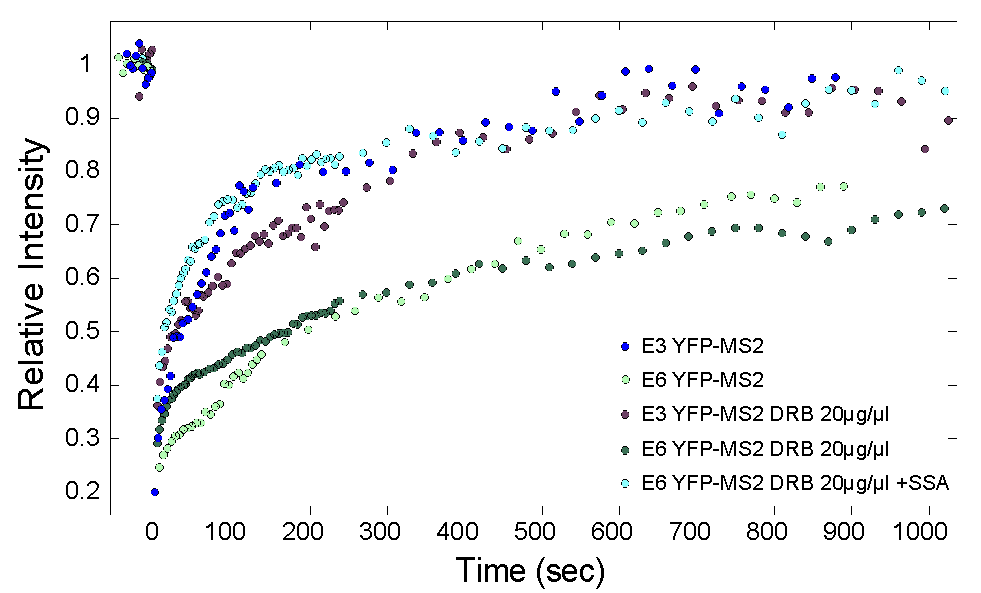

Supplement: Figure S11 — Low DRB does not affect kinetics on E3 and E6 genes. No change in the FRAP recovery kinetics of YFP-MS2 on cells treated with DRB (20 µg/µl for 2 h before imaging): E3 cells (brown), E6 cells (dark green), or E6 cells with SSA (cyan) SSA; compared to untreated E3 (blue) and E6 (light green) cells. (0.14 MB TIF) [file pbio.1000573.s011.tif]
